# A nano-liposome formulation of the PARP inhibitor Talazoparib enhances treatment efficacy and modulates immune cell populations in mammary tumors of BRCA-deficient mice

**DOI:** 10.7150/thno.36281

**Published:** 2019-08-14

**Authors:** Di Zhang, Paige Baldwin, Ana S. Leal, Sarah Carapellucci, Srinivas Sridhar, Karen T. Liby

**Affiliations:** 1 Michigan State University, East Lansing, MI, USA; 2 Northeastern University, Boston, MA, USA; 3 Harvard Medical School, Boston, MA, USA

**Keywords:** PARP inhibitor, Talazoparib, Nanoparticle, BRCA-deficient breast cancer, immunomodulation

## Abstract

Two recently approved PARP inhibitors provide an important new therapeutic option for patients with BRCA-mutated metastatic breast cancer. PARP inhibitors significantly prolong progression-free survival in patients, but conventional oral delivery of PARP inhibitors is hindered by limited bioavailability and off-target toxicities, thus compromising the therapeutic benefits and quality of life for patients. Here, we developed a new delivery system, in which the PARP inhibitor Talazoparib is encapsulated in the bilayer of a nano-liposome, to overcome these limitations.

**Methods**: Nano-Talazoparib (NanoTLZ) was characterized both *in vitro* and *in vivo*. The therapeutic efficacy and toxicity of Nano-Talazoparib (NanoTLZ) were evaluated in BRCA-deficient mice. The regulation of NanoTLZ on gene transcription and immunomodulation were further investigated in spontaneous BRCA-deficient tumors.

**Results**: NanoTLZ significantly (p<0.05) prolonged the overall survival of BRCA-deficient mice compared to all of the other experimental groups, including saline control, empty nanoparticles, and free Talazoparib groups (oral and i.v.). Moreover, NanoTLZ was better tolerated than treatment with free Talazoparib, with no significant weight lost or alopecia as was observed with the free drug. After 5 doses, NanoTLZ altered the expression of over 140 genes and induced DNA damage, cell cycle arrest and inhibition of cell proliferation in the tumor. In addition, NanoTLZ favorably modulated immune cell populations *in vivo* and significantly (p<0.05) decreased the percentage of myeloid derived suppressor cells in both the tumor and spleen compared to control groups.

**Conclusions**: Our results demonstrate that delivering nanoformulated Talazoparib not only enhances treatment efficacy but also reduces off-target toxicities in BRCA-deficient mice; the same potential is predicted for patients with BRCA-deficient breast cancer.

## Introduction

Mutations in breast cancer-associated (*BRCA*) genes are the leading cause of hereditary breast cancer. Women with *BRCA* mutations have up to an 80% lifetime risk of developing breast cancer 1. Furthermore, the majority of *BRCA1* mutated tumors are basal-like 2, a subtype associated with poor prognosis 3. *BRCA1* functions as an important tumor suppressor that acts as a gatekeeper to protect against genomic instability 4. Besides regulating numerous cellular functions 5, 6, including cell cycle, apoptosis and transcription, the BRCA1 protein is essential for repairing double-stranded DNA breaks through the homologous recombination (HR) pathway. Loss of function of the *BRCA1* gene forces cells to rely on more error-prone mechanisms such as non-homologous end joining to repair DNA damage 7. PARP (poly (ADP-ribose) polymerase) is another protein that plays critical roles in DNA repair. PARP1 recognizes single-stranded DNA breaks and recruits proteins to assemble and activate DNA base excision repair machinery 8. Single-stranded DNA breaks that cannot be repaired because of PARP deficiency will propagate into double-stranded DNA breaks and need to be repaired by the BRCA-initiated HR pathway. If the BRCA protein is dysfunctional, PARP is necessary for proper DNA repair. Because of the interdependence between PARP and BRCA, PARP inhibitors were developed to treat BRCA-deficient cancers by inducing synthetic lethality 9, 10. Synthetic lethality occurs when a perturbation in either of two genes does not reduce cell survival, but the simultaneous perturbation of both genes results in a loss of cell viability. In the case of a *BRCA* mutation, PARP inhibitors block the repair of single-strand DNA breaks, which are then converted into double-strand breaks during replication. Because the cell cannot proceed through homologous recombination, it must rely on error prone pathways which results in genomic instability and cell death.

Before PARP inhibitors were developed, no targeted therapy was available for patients with BRCA mutations who developed breast cancer. These tumors do not usually express estrogen, progesterone or HER2 receptors, so cytotoxic chemotherapy remained the standard of care for these mostly triple negative breast cancer (TNBC) patients. In January 2018, Olaparib became the first PARP inhibitor approved by the FDA for treating germline BRCA-mutated metastatic breast cancer. In October 2018, after the Phase 3 EMBRACA clinical trial showed that Talazoparib significantly extended progression-free survival in patients with metastatic breast cancer, Talazoparib was approved. This second PARP inhibitor to market was approved for treating germline BRCA-mutated, HER2-negative breast cancer, either locally advanced or metastatic 11. Talazoparib is approximately 100 times more potent than Olaparib because of its higher capacity to induce “PARP trapping” 12. Talazoparib not only inhibits the catalytic activity of PARP but also traps PARP at the site of DNA damage, thus inducing cell death. Notably, in addition to mutations in *BRCA1/2* genes, a wide range of other mechanisms produce a similar phenotypic trait of HR deficiency, which is called “BRCAness” 13. The loss of HR can be the result of epigenetic silencing of *BRCA1/2* genes or genetic alterations in other key players such as *RAD51, ATR, CHK1/2, ATM, FANCD2* and *FANCA* along the HR pathway 14. Recent studies reported that *MYC* amplification, *p53* mutations, and loss of *PTEN* all contribute to a BRCA-like behavior 15, 16. Therefore, PARP inhibitors could potentially impact more breast cancer patients beyond populations with *BRCA* mutations. A diverse collection of triple negative breast cancer patient-derived xenografts has confirmed the therapeutic activity of PARP inhibitors even in *BRCA1/2* wild-type tumors 17.

Currently, PARP inhibitors are formulated for daily oral administration based on their pharmacokinetic profiles. However, the bioavailability of Talazoparib is only 56% in rats, meaning a higher dose must be administered systemically to achieve the desired effect 18. Talazoparib is the most potent PARP inhibitor, but this potency also increases side effects such as alopecia, fatigue, anemia, thrombocytopenia, neutropenia and decreased appetite 19. Myelodysplastic syndrome, myelosuppression and embryo-fetal toxicity are included as warnings and precautions on the package insert. One method to overcome poor bioavailability and subsequent toxicity is the use of nanoparticle delivery systems.

Nanoparticles as drug delivery systems aim to increase bioavailability, minimize drug metabolism upon administration, prevent side effects, and increase the amount of drug delivered to the desired target 20. Additionally, nanocarriers take advantage of the enhanced permeability and retention effect, in which tumors rapidly generate blood vessels that are considered “leaky” allowing for particles to extravasate and accumulate in the tumor microenvironment 21. Liposomes are self-assembled phospholipid bilayers that form vesicles and are biologically inert and biocompatible. Due to the similar morphology to cell membranes and ability to incorporate both hydrophilic and lipophilic compounds, liposomes are thought to be ideal nanocarriers 22. Although liposome efficacy can be limited by uptake into the reticuloendothelial system (RES), polyethylene glycol (PEG) can be fused to the surface to extend the circulation time by conferring “stealth” properties to bypass some of the uptake by RES organs 23. Here, we encapsulated Talazoparib in the bilayer of a nano-liposome and evaluated the efficacy and toxicity of Nano-Talazoparib (NanoTLZ) compared to free drug in BRCA-deficient mice.

## Materials and Methods

### Synthesis of NanoTLZ

Talazoparib for all experiments was purchased from Selleck Chemicals (Catalog # S7048, purity: 99.8%). NanoTLZ was synthesized using 1, 2-dipalmitoyl-*sn*-glycero-3-phosphocholine (DPPC), 1,2-dioleoyl-3-tri methyl-ammonium-propane (chloride salt) (DOTAP), cholesterol, 1,2-distearoyl-*sn*-glycero-3 phosphoethanolamine-N-[methoxy(polyethyleneglycol)-2000 (DSPE-PEG_2000_, Avanti Polar Lipids), and Talazoparib. DOTAP was incorporated within the formulation because some literature suggests it can enhance the encapsulation of hydrophobic compounds 24, 25. It also imparts a slightly positive surface charge, which allows better cell uptake, as cells more readily take up positively charged particles. DPPC, cholesterol, DOTAP and DSPE-PEG_2000_, were individually dissolved in a methanol/ethanol mixture at a molar ratio of 65:29:2:4. 11.17 mM Talazoparib was added in dimethylformamide. Nanoparticles were formed via nanoprecipitation using the NanoAssemblr Benchtop. The total flow rate was 4 ml/min, and the aqueous to organic flow rate was 3:1. Organic solvents were removed by evaporation under argon followed by dialysis against phosphate buffered saline (PBS) for 30 minutes. The non-encapsulated drug which is insoluble in aqueous media was removed via syringe filter 26, and validation of the removal of free drug is shown in Figure S1. Encapsulation efficiency was calculated using the formula (C_final_/C_initial_) x100, where C_final_ is the concentration of drug in 1 ml of particles after processing and C_initial_ is the concentration of drug added to prepare 1 ml of particles. Drug loading was calculated using the following formula where C_final_ is the concentration of drug in 1 ml of particles after processing and W_feed_ is the weight of lipids in the feed to prepare 1 ml of formulation: drug loading (%) = C_final_/W_feed_ x 100. Drug release was characterizd previously 27. Vehicle nanoparticles (empty nanoparticles) were prepared following the same protocol without the addition of Talazoparib. Cyanine 5 (Cy5) labeled particles were synthesized by addition of Cy5 dye in the lipid mixture.

### Characterization of NanoTLZ

The size and zeta potential of the nanoparticles was measured using a Brookhaven 90Plus analyzer equipped with ZetaPALS. Nanoparticles were diluted 1:100 in 0.2X PBS for all measurements. Stability was assessed by measuring size and zeta potential at predetermined times over the course of 2 months. The size was confirmed by transmission electron microscopy using a negative stain of 1.0% uranyl acetate. The concentration of encapsulated Talazoparib was measured via high performance liquid chromatography (HPLC) following nanoparticle lysis with methanol. HPLC was performed on an Agilent 1260 Infinity II instrument with a reverse phase C18 Supelco column. The mobile phase A consisted of acetonitrile with 0.1% phosphoric acid and the mobile phase B consisted of water with 0.1% phosphoric acid. The following gradient was applied 10-95% A (0-5.3 min), 95% A (5.3-8.5 min), 95-10% A (8.5-10.0 min), 10% A (10-11.5 min). The flow rate was 0.82 ml/minute, and Talazoparib was detected at a wavelength of 309 nm at ~4.2 minutes.

### Cell Culture

**Figure 1:**
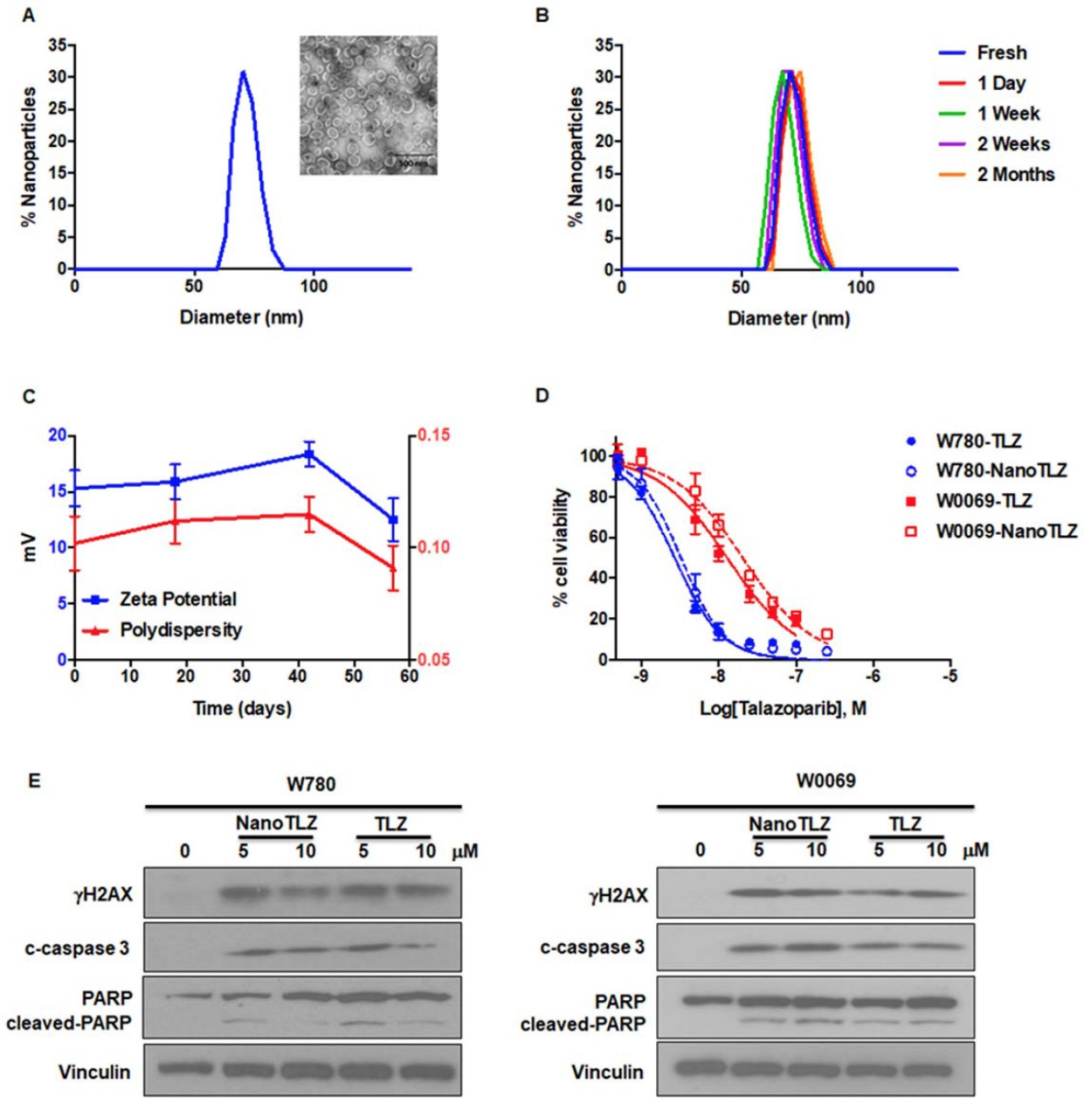
Characterization of Nano-Talazoparib (NanoTLZ). A. Physicochemical characterization of NanoTLZ via dynamic light scattering and (inset) transmission electron microscopy after staining with 1.0% uranyl acetate illustrates a monodisperse formulation with an average diameter of 75 nm. B and C. NanoTLZ is stable in size, zeta potential and polydispersity, for up to 2 months in storage at 4°C. D. W780 and W0069 cells were treated with NanoTLZ or free Talazoparib (TLZ) for 6 days. Cell viability was detected by the MTS assay. E. W780 and W0069 cells were treated with NanoTLZ or free Talazoparib (TLZ) for 48 hrs. NanoTLZ and TLZ increased the expression of γH2AX, cleaved-caspase 3 (c-caspase 3) and cleaved-PARP in these BRCA-deficient breast cancer cells.

W0069 and W780 cells derived from mammary tumors of BRCA-deficient mice were provided by Dr. Chu-Xia Deng (National Institutes of Health, Bethesda, MD) 28 and were cultured in DMEM+10% FBS+1% Pen/Strep (Corning Cellgro, Mediatech, Manassas, VA). In a dose response assay (Figure 1), cells were seeded into 96 well plates at 1000 cells per well. The following day cells were exposed to either Talazoparib or NanoTLZ at concentrations ranging from 0-100 nM. One week after seeding, cell viability was ascertained by the MTS assay to measure the metabolic activity of the cells. Data from the dose response experiment were plotted and fit using a variable slope four-parameter logistic equation constrained at 100 and 0. In the biomarker assay (Figure 1E), cells were seeded in 6 well plates at 150,000 cells per well. The next day, cells were treated with Talazoparib or NanoTLZ at 5 and 10 μM. After 48 hrs of treatment, cells were harvested and proteins were extracted for western blotting.

### Pharmacokinetics and Pharmacodynamics

All animal studies were performed in accordance with protocols approved by the Institutional Animal Care and Use Committees (IACUC) at Michigan State University and Northeastern University. An orthotopic xenograft model of human BRCA-mutated breast cancer was established via injection of 5x10^6^ HCC1937 cells (ATCC, Manassas, VA; cells were cultured in RPMI1640 with 10% FBS and 1% Pen/Strep) in the mammary fat pad of female NCr-nu/nu mice. Mice with tumors ~100 mm^3^ in size were administered a single dose of 1 mg/kg i.v. NanoTLZ. Mice were euthanized at designated time points for sample collection. Blood was collected via cardiac puncture into K2 EDTA microtainers. Blood was centrifuged at 1600 *g* for 15 minutes at 4°C. Plasma was separated and frozen at -80°C until processed. Acetonitrile was added to precipitate plasma proteins. Samples were centrifuged at 14,000 *g* for 5 minutes, and the supernatant was filtered with a 0.2 μm syringe filter. Each sample was dried overnight and reconstituted in 200 µl of 50:50 methanol:water solution for analysis via HPLC. HPLC conditions were as detailed above. A standard curve was prepared by processing plasma from untreated animals and spiking the samples with known amounts of Talazoparib when reconstituting the samples. A two compartment model was fit using PKSolver 29.

Tumors were collected and snap frozen in liquid nitrogen to stabilize PAR levels. Tumors were minced in lysis buffer (1% w/w deoxycholic acid, 1% w/w triton-x, 0.1% w/w sodium lauryl sulfate) with protease inhibitors (1 mM PMSF, 1 mM AEBSF•HCL, 800 nM aprotinin, 50 µM bestatin, 15 µM E-64, 5 mM EDTA, 20 µM leupeptin, and 10 µM pepstatin A). Total protein content was determined using the BCA assay (Pierce). Levels of PAR in the tumor lysates were determined by ELISA using PARP in vivo PD Assay II kit (Trevigen) following the manufacturer's instructions.

Brca1^Co/Co^;MMTV-Cre;p53^+/-^ mice bearing tumors (N=4) were injected i.v. with 100 μl nanoparticles encapsulated with the fluorescent dye Cy5. Mice were imaged using an IVIS imaging system (PerkinElmer) 24 hrs after the injection. Auto exposure setting was used to acquire the pictures. Major organs were dissected and imaged for biodistribution after whole body imaging.

To evaluate the cellular uptake of nanoparticles, W780 cells were plated in 12-well plates. Cells were treated with either 5% empty nanoparticles or 5% nanoparticles encapsulated with the fluorescent dye Cy5 for 1-2 hrs. Cells were then fixed with 10% neutral buffered formalin (Sigma-Aldrich) for 15 minutes and mounted with Gold Antifade Mountant with DAPI (Invitrogen). Cy5 fluorescence (Green channel) and DAPI (UV channel) were detected using a fluorescence microscope (Nikon TE2000-U Inverted Microscope).

### *In Vivo* Treatment Studies

The therapeutic efficacy of NanoTLZ was assessed in Brca1^Co/Co^;MMTV-Cre;p53^+/-^ mice 30, 31. Treatment was started when a tumor was 4-5 mm in diameter and ended when it reached 10 mm in diameter. Mice with established tumors were randomized into five treatment groups: control (saline, i.v. N=5), empty nanoparticles (i.v., N=5), NanoTLZ (i.v., 0.33 mg/kg, N=8), free Talazoparib (i.v., 0.33 mg/kg, N=8), and free Talazoparib (gavage, 0.33 mg/kg, N=8). NanoTLZ was diluted in saline to the working concentration. Free Talazoparib was dissolved in DMSO and diluted in saline (DMSO in the final solution was 1%). Treatment was given three times a week (M, W, F). All the mice were weighed before each injection. Tumor size was measured using a caliper twice a week.

### Western Blotting

W780 and W0069 cells treated with Talazoparib or NanoTLZ were lysed in RIPA buffer (1 M Tris-Cl, 5 M NaCl, pH 7.4, 0.5 M EDTA, 25 mM deoxycholic acid, 1% triton-X, 0.1% SDS) with protease inhibitors (1 mM PMSF, 2 µg/ml aprotinin and 5 µg/ml leupeptin). Tumor samples dissected from BRCA-deficient mice were homogenized and lysed in EBC buffer (5 mol/L NaCl, 1 mol/L Tris pH 8) with protease inhibitors and 10% NP-40. Protein concentrations were determined using the BCA assay (Sigma-Aldrich). 20 µg of protein were separated by 10% SDS-PAGE gels and transferred to nitrocellulose membranes. γH_2_AX (Abcam, 1:1000), cleaved-caspase 3 (Cell Signaling, 1:1000), PARP/cleaved-PARP (Cell Signaling, 1:1000), Cyclin D1 (Cell Signaling, 1:1000), Cyclin E1 (Cell Signaling, 1:1000), PCNA (Santa Cruz, 1:1000), and vinculin (Cell Signaling, 1:4000) primary antibodies were used to detect the corresponding proteins. Secondary antibodies (anti-rabbit or anti-mouse linked to HRP) were purchased from Cell Signaling. ECL Western blotting substrate (GE Healthcare Life Sciences, UK) was used to detect the signal. Images shown are representative of 3 independent experiments. ImageJ was used to quantify protein expression.

### Immunohistochemistry

Brca1^Co/Co^;MMTV-Cre;p53^+/-^ mice were treated with five doses (three times a week) of saline, i.v.TLZ or NanoTLZ (N=5/group). Tumor, mammary gland and spleen were then harvested and sectioned for histopathology and immunohistochemistry. EDTA (Cell Signaling, for CD3) or citrate buffer (Vector, Cat. # H3300, for all the other antibodies) was used for antigen retrieval. Endogenous peroxidase activity was quenched using hydrogen peroxide (3%) for 10 minutes. Sections were stained with CD45 (1:100, BioScience), CD3 (1:40, Biolegend), Gr-1 (1:50, R&D), F4/80 (1:50, Invitrogen), Foxp3 (1:25, BioScience), PCNA (1:200, Santa Cruz), or γH_2_AX (1:100, Abcam) antibodies. Anti-rat secondary antibody was purchased from Vector. Anti-mouse and anti-rabbit secondary antibodies conjugated to HRP were purchased from Cell Signaling. Signal was detected using a DAB kit (Cell Signaling). Sections were counterstained with hematoxylin (Vector).

### Flow cytometry

Brca1^Co/Co^;MMTV-Cre;p53^+/-^ mice (N=5/group) with established tumors (4-5 mm in diameter) were treated for five doses (three times a week) and then tumor, spleen and mammary gland were collected and digested for flow cytometry as published previously 32. Panel 1: CD45-VioGreen (Miltenyi, 3 μg/mL), Gr-1-PE (Miltenyi, 3 μg/mL), CD11b-FITC (Miltenyi, 3 μg/mL), CD19-PerCP/Cy5.5 (BioLegend, 2 μg/mL). Panel 2: CD45-VioGreen (Miltenyi, 3 μg/mL), CD4-FITC (Miltenyi, 3 μg/mL), CD3-PE (BioLegend, 2 μg/mL), CD8-APC (BioLegend, 2 μg/mL), CD25-PE/Cy7 (BioLegend, 2 μg/mL).

### RNAseq

Tumors from Brca1^Co/Co^;MMTV-Cre;p53^+/-^ mice treated with saline, free Talazoparib (i.v.), or NanoTLZ for five doses (three times a week) were harvested. Three samples were collected for each group. Total RNA was isolated using the RNeasy Mini Kit (Qiagen, Valencia, CA). The RNA integrity number (RIN) was detected using the Aligent Bioanalyzer at the MSU Research Technology Support Facility (RTSF) Genomics Core facility. RNAseq and the bioinformatics analysis were performed by Novogene (Sacramento, CA) as described in a previous publication 32. Raw data and processed data were deposited on Gene Expression Omnibus (GEO) and are accessible through GSE125206.

### RT-qPCR analysis

To validate the RNAseq data, aliquots of RNA samples from the tumor were used to run RT-qPCR analysis. RNA concentrations were determined by NanoDrop, and 2 µg RNA was used to synthesize cDNA using SuperScript III reverse transcriptase (Invitrogen, Carlsbad, CA). Primers (sequences shown in Table S1) were ordered from IDT, except Cxcl12. Validated and optimized primers for Cxcl12 were purchased from Qiagen (Valencia, CA). iQ SYBR Green Supermix (Bio-Rad, Berkeley, CA) and the QuantStudio 7 Flex Real-Time PCR system were used to detect gene expression. The delta-delta Ct method was used to assess relative gene expression 33. Values were normalized to the reference gene GAPDH and expressed as fold change compared to saline control samples.

### Statistical Analysis

The *in vitro* experiments were performed in triplicate, and independent experiments were repeated at least three times. Results were expressed as mean ± SEM. For the *in vivo* experiments, results were analyzed using one-way ANOVA followed by a Tukey test if the data fit a normal distribution; the Kruskal-Wallis one-way ANOVA on ranks was used followed by the Dunn test for multiple comparisons if the data did not fit a normal distribution (Prism 6). A paired t-test was used to compare body weight before and after treatment. The log-rank (Mantel-Cox) test was used to compare survival curves. For the growth of tumors, a Chi-Square test was used to compare proportions. p<0.05 was considered statistically significant. For the RNAseq analysis, differential expression analysis was performed using the DESeq2 R package 34, and padj<0.05 was considered statistically significant.

## Results

### Validation of NanoTLZ *in vitro* and *in vivo*

To overcome the limitations of oral delivery, we developed a new formulation of Talazoparib. We encapsulated Talazoparib into liposomal nanoparticles with an average size of 74.5 ± 11.0 nm (Figure 1A). The encapsulation efficiency was 83.0±5.9% and drug loading was 1.0 ± 0.1%. NanoTLZ is stable in size and zeta potential (15.3 ± 1.6 mV) at 4°C for 2 months (Figure 1B-C). To validate the effects of NanoTLZ *in vitro*, BRCA-deficient cancer cell lines (W780 and W0069) were treated with NanoTLZ and free Talazoparib. Both W780 and W0069 cell lines were derived from independent tumors that developed in BRCA-deficient mice. W780 cells were derived from a tumor classified as an adenocarcinoma, while W0069 cells were derived from a fibro-adenoma tumor with strong stromal reaction 30. NanoTLZ was similar in potency to free Talazoparib in both cell lines (Figure 1D). Empty nanoparticles (vehicle) had no effects on cell viability (Figure S2). As reported previously for PARP inhibitors 35, NanoTLZ and Talazoparib induced DNA damage and apoptosis, as indicated by an increase of γH2AX, cleaved-caspase 3 and cleaved-PARP, respectively, in W780 and W0069 cells (Figure 1E).

**Figure 2:**
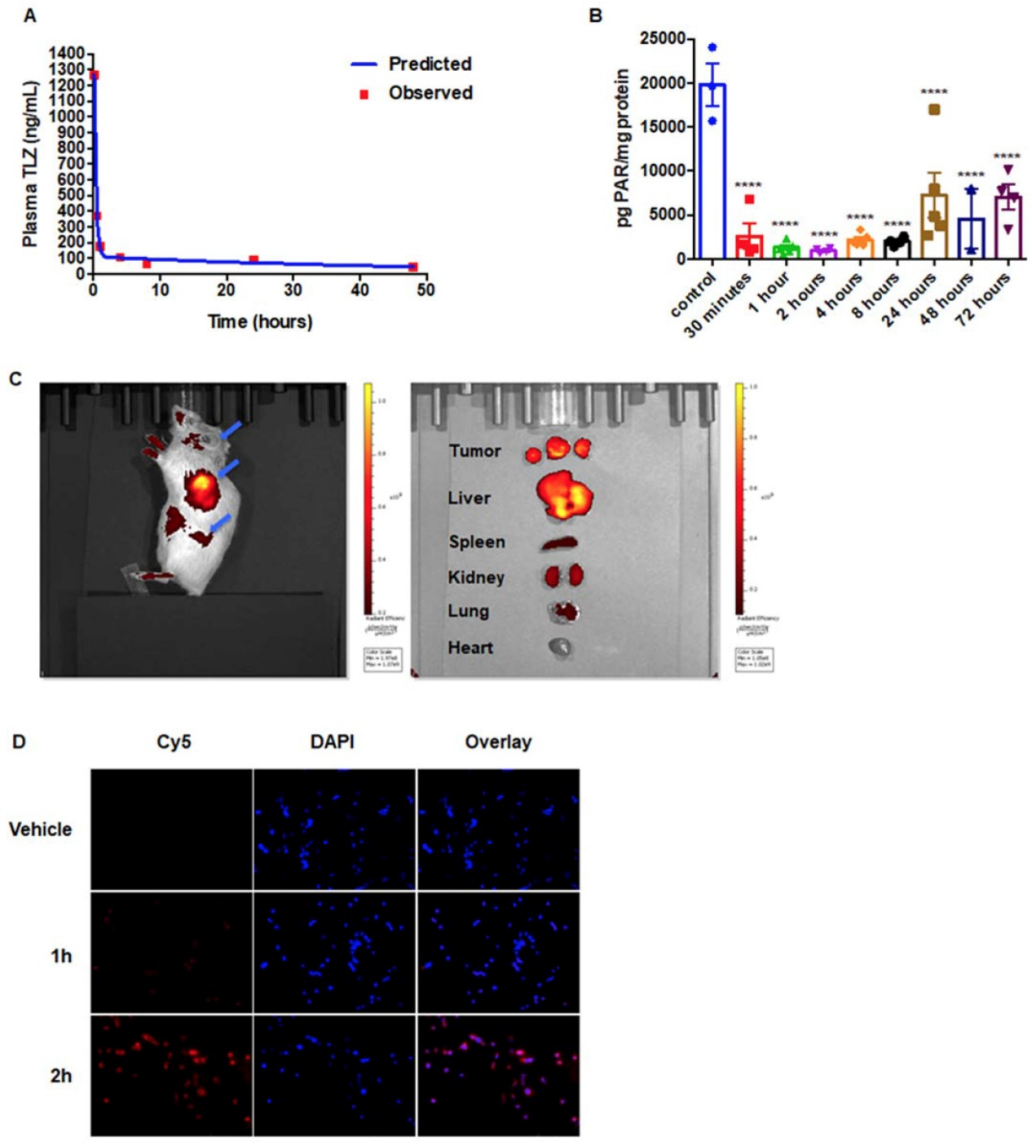
Pharmacokinetics and pharmacodynamics of NanoTLZ. A. Mice bearing orthotopic BRCA-mutated human HCC1937 xenografts were injected with 1 mg/kg NanoTalazoparib (i.v.). Plasma drug concentrations were detected via HPLC. A two-compartment model was fit to the plasma data using PKSolver. B. Tumor PAR levels were detected via ELISA. ****, p<0.0001 for all time points compared to control C. Brca1^Co/Co^;MMTV-Cre;p53^+/-^ mice (N=4) were injected with a single dose of Cy5-encapsulated nanoparticles, and the fluorescent signal was detected using a IVIS spectrum imaging system 24 hrs after injection. Major organs were dissected after *in vivo* imaging (left) and biodistribution of nanoparticles in these organs was detected (right). Blue arrows point to tumors. Representative images are shown. D. W780 cells were treated with 5% empty nanoparticles (vehicle) or Cy5-encapsulated nanoparticles for 1-2 hrs. Fluorescent Cy5 signal was detected with a fluorescence microscope. 200x magnification.

To evaluate the pharmacokinetics of NanoTLZ, an orthotopic xenograft model of human BRCA-mutated HCC1937 cells was established. Tumor bearing mice were dosed with 1 mg/kg NanoTLZ i.v., then blood and tumor samples were collected at different time points. The plasma data fits a two-compartment model with a terminal half-life of 37.5 hours (Figure 2A). PAR levels, a marker of PARP activity, were detected in the tumor samples. The PAR level was significantly (p<0.0001) decreased 30 mins after injection and remained at lower levels than control tumors up to 72 hours post injection (Figure 2B).

To validate the enhanced permeability and retention effects of nanoparticles in our Brca1^Co/Co^;MMTV-Cre;p53^+/-^ model, we encapsulated the fluorescent dye Cy5 into nanoparticles using the same method as for Talazoparib and injected them i.v. into tumor-bearing BRCA-deficient mice. Mice and major organs were imaged using an IVIS system. At 24 hrs after injection, the fluorescent signal was mainly in the tumor, suggesting preferential accumulation in the tumor, although signal was also detected in the liver (Figure 2C). In addition, the cellular uptake of nanoparticles was assessed in W780 cells *in vitro*. Cells were treated with 5% nanoparticles encapsulated with Cy5, and the fluorescent Cy5 signal was detected using a fluorescence microscope. Nanoparticles were visible inside of cells within 2 hours (Figure 2D).

### Nano-formulation enhances the efficacy of Talazoparib and reduces the toxicity in BRCA-deficient mice

**Figure 3:**
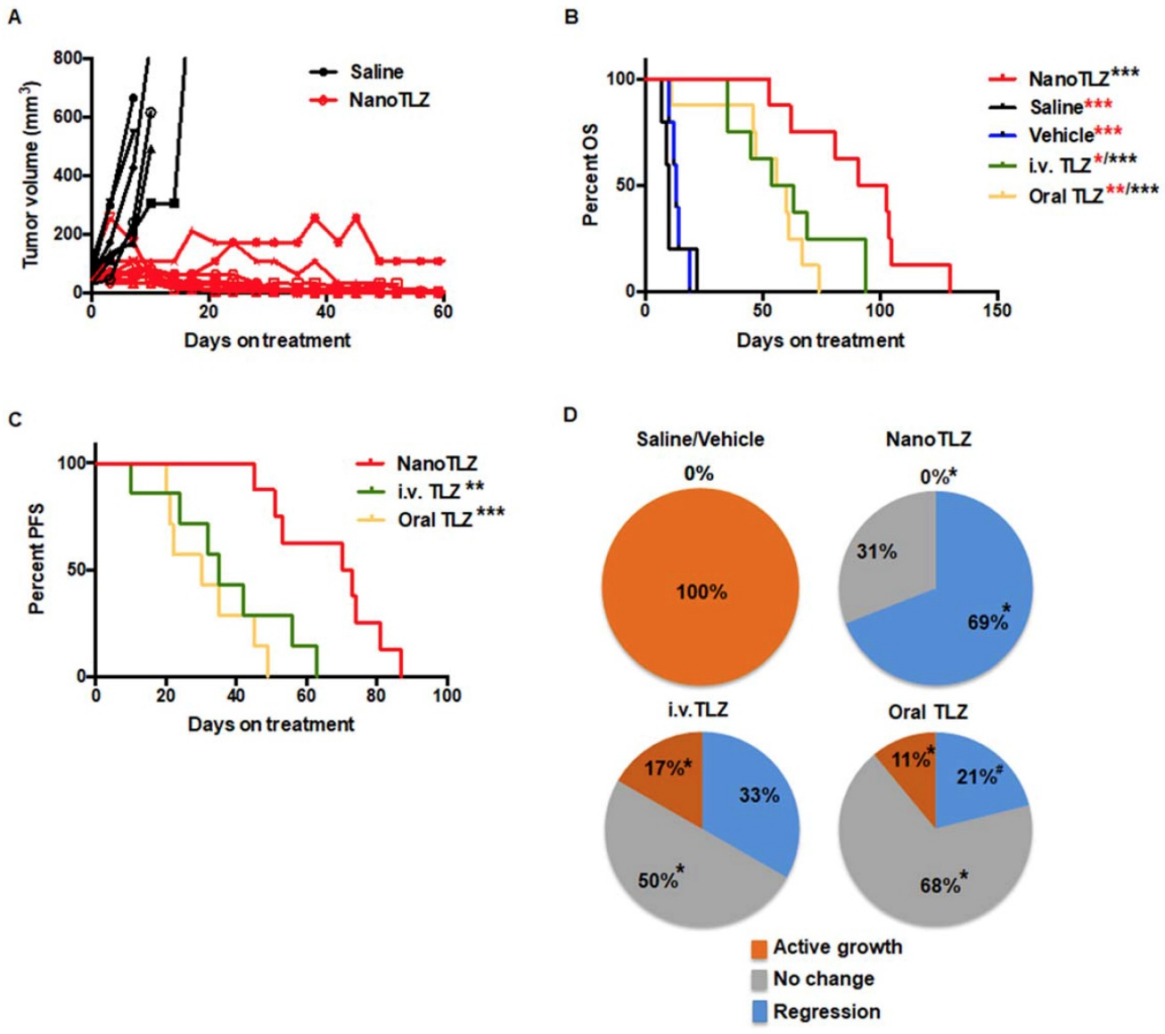
NanoTLZ prolonged the overall survival and was more effective in inducing tumor regression compared to free Talazoparib in BRCA-deficient mice. Brca1^Co/Co^;MMTV-Cre;p53^+/-^ mice were started on treatment when tumors were 4 mm in diameter. The treatments were either control (saline, i.v.), empty nanoparticles (vehicle, i.v.), NanoTLZ (i.v., 0.33 mg/kg), free Talazoparib (i.v.TLZ, i.v., 0.33 mg/kg), or free Talazoparib (oral TLZ, gavage, 0.33 mg/kg). Treatment was given three times a week. Mice were sacrificed when the tumor reached 10 mm in diameter. A. Growth curves of individual tumors treated with saline or NanoTLZ. (N= 6 in saline and 9 in NanoTLZ groups) B. NanoTLZ significantly prolonged the overall survival of BRCA-deficient mice compared to controls, oral TLZ and i.v. TLZ. N=5 in saline and vehicle groups, N=8 in NanoTLZ and TLZ treatment groups. Symbols in red: *, p<0.05, **, p<0.0.1, ***, p<0.001 vs. NanoTLZ. Symbols in black: ***, p<0.001 vs. saline. C. NanoTLZ significantly improved the progression free survival of BRCA-deficient mice compared to i.v. TLZ and oral TLZ. N=8. **, p<0.0.1, ***, p<0.001 vs. NanoTLZ. D. All tumors were classified into three groups: regressing (tumor volume decreased more than 50%), no change (tumor volume did not increase or decrease by more than 50%), active growth (tumor continued to grow and tumor volume increased more than 50%). N=6-19/group. *, p<0.05. vs. saline; ^#^, p<0.05 vs. NanoTLZ.

Because of the tumor specificity of nanoparticles, we hypothesized that NanoTLZ would enhance the efficacy of Talazoparib and be more tolerable for treating BRCA-deficient breast cancer. When tumors in Brca1^Co/Co^;MMTV-Cre;p53^+/-^ mice reached 4 mm in diameter, they were randomized and enrolled into five experimental groups: saline (i.v.), vehicle (empty nanoparticle, i.v.), NanoTLZ (0.33 mg/kg, i.v.), free Talazoparib (0.33 mg/kg, i.v.) or free Talazoparib (0.33 mg/kg, oral). Mice were treated three times a week (M, W, F) until a tumor reached 10 mm in diameter. All tumors in the saline control group grew exponentially (Figure 3A) and lived an average of only 11.6±2.7 days once treatment started (Figure 3B). Similarly to the saline treated group, empty nanoparticles did not have any therapeutic effect with overall survival in this group of 13.6±1.5 days. Drug treatment by all routes significantly (p<0.05) prolonged the overall survival (range 52.8-91.1 days) compared to the saline control, which validated the effectiveness of Talazoparib in treating BRCA-deficient tumors. There was no statistical significance observed between oral free Talazoparib and i.v. free Talazoparib in either overall survival (Figure 3B) or progression-free survival (Figure 3C). Progression was defined as a 50% increase in tumor volume. However, NanoTLZ treatment rapidly induced tumor regression (Figure 3A) and significantly (p<0.05) prolonged both overall survival and progression-free survival compared to all of the other groups, including the free drug treatment groups (Figure 3B-C). The average life span once treatment started was extended from 52.8±6.8 days in the oral free Talazoparib group and 61.1±8.3 days in the i.v. free Talazoparib group to 91.1±8.9 days in the NanoTLZ group. NanoTLZ was able to maintain its effectiveness longer than the free Talazoparib treatment.

Furthermore, NanoTLZ was also more effective than free Talazoparib in inducing tumor regression in BRCA-deficient mice. Tumors were classified into three categories: active growth (>50% increase in tumor volume), no change (<50% change in tumor volume), and regression (>50% decrease in tumor volume). Tumors in the control groups (saline and empty nanoparticles) were all actively growing. All of the tumors treated with NanoTLZ responded (p<0.05) to the treatment with no active growth (0%) and 69% (9 out of 13) regressed after treatment (Figure 3D). In comparison, 17% (2 out of 12) of tumors in the i.v. Talazoparib group and 11% (2 out of 19) in the oral Talazoparib group did not respond to the treatment and continued to grow (Figure 3D). The majority of the tumors in these two groups achieved disease stabilization as only 33% (4 out of 12) tumors in the i.v. Talazoparib group and 21% (4 out of 19) in the oral Talazoparib group regressed (Figure 3D). Notably, among the tumors that regressed, 56% (5 out of 9) tumors could no longer be palpated in the NanoTLZ group, while only 25% (1 out of 4) of the mice were tumor-free in the i.v. Talazoparib group and none (0 out of 4) were tumor-free in the oral Talazoparib group.

**Figure 4:**
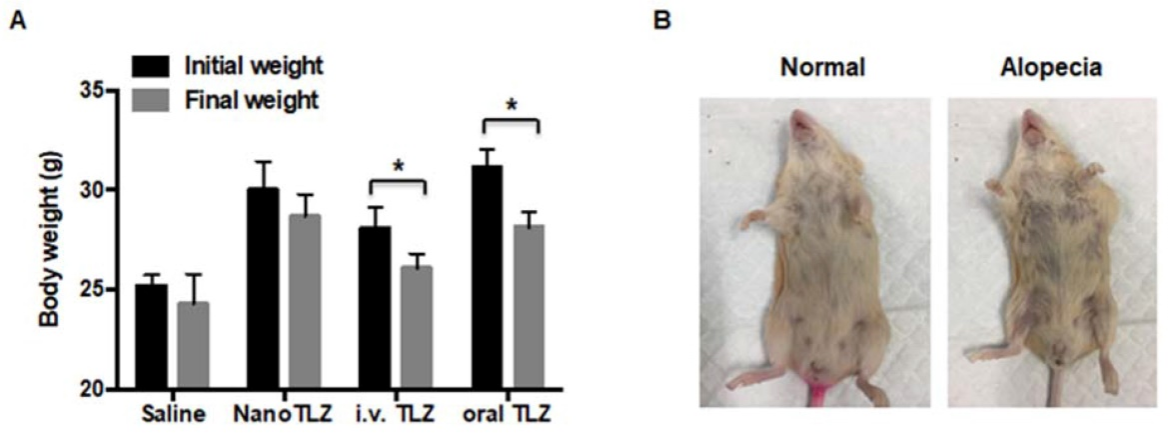
NanoTLZ is better tolerated than free Talazoparib in BRCA-deficient mice. A. Brca1^Co/Co^;MMTV-Cre;p53^+/-^ mice were treated three times a week and weighed prior to each treatment. Initial weight, the weight when the mice were started on treatment; final weight, weight after 10 injections of the corresponding treatment. Data presented as mean ± SEM. N=5 in saline group and N=8 in the NanoTLZ and Talazoparib treatment groups (i.v. TLZ and oral TLZ). *, p<0.05 vs*.* initial weight. B. Representative pictures of alopecia observed in free Talazoparib treatment groups.

All mice were weighed immediately before each injection. Body weight was significantly (p<0.05) decreased in both the i.v. free Talazoparib (from 28.1±1.1 g to 26.1±0.7 g) and oral Talazoparib (from 31.1±0.9 g to 28.1±0.8 g) treatment groups after 10 doses (Figure 4A). In contrast, NanoTLZ was better tolerated with no significant changes in body weight (from 30.0±1.4g to 28.6±1.1g, Figure 4A). The dynamic changes in body weight over time are shown in Figure S3. Additionally, alopecia, a known side effect of Talazoparib in patients, was observed in both the i.v. Talazoparib (2 out of 8) and oral Talazoparib groups (2 out of 8), but no alopecia was observed in any of the mice in the NanoTLZ treatment group (0 out of 8). A representative image of alopecia is shown in Figure 4B. No liver toxicity was observed after long-term treatment of NanoTLZ, based on the normal histology of all of the liver tissues (Figure S4).

### RNAseq analysis of BRCA-deficient tumors treated with NanoTLZ and i.v. Talazoparib

**Figure 5:**
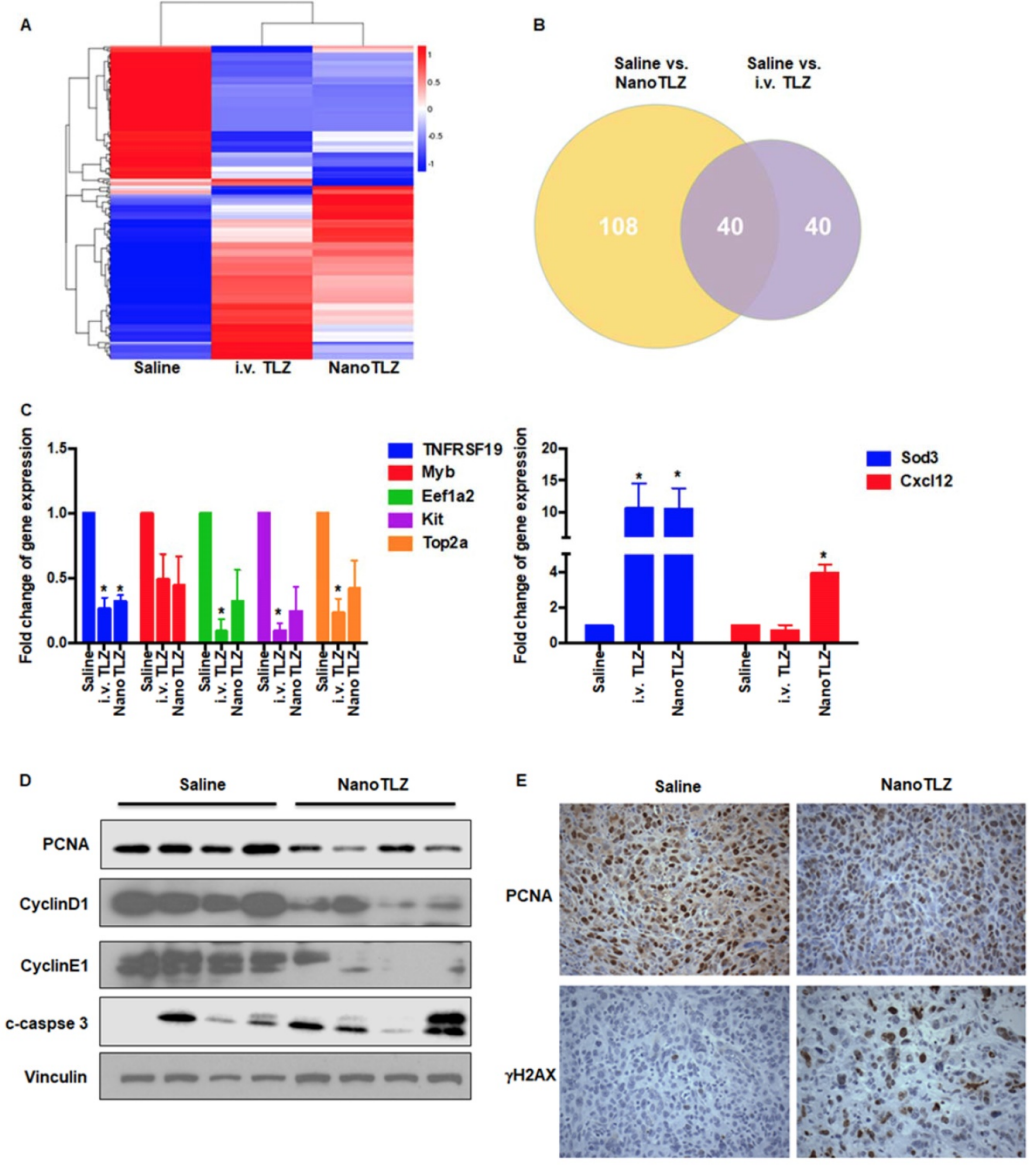
RNAseq analysis of tumors from BRCA-deficient mice treated with NanoTLZ or i.v. Talazoparib. When Brca1^Co/Co^;MMTV-Cre;p53^+/-^ mice developed tumors 4 mm in diameter, they were treated with either saline, NanoTLZ (0.33 mg/kg), or Talazoparib (i.v. TLZ, 0.33 mg/kg) by i.v. for 5 doses (3 times a week). Total RNA was isolated from the tumors and processed for RNAseq analysis. A. Cluster analysis of differentially expressed genes in each group. N=3 mice/group. B. Venn diagram of differentially expressed genes in NanoTLZ and i.v. Talazoparib group. C. Validation of gene expression by real-time PCR. Data presented as mean ± SEM. N=5 mice/group. *, p<0.05 vs. Saline. D. Protein expression of PCNA, CyclinD1, CyclinE1, cleaved-caspase (c-caspase) 3 in tumors treated with saline or NanoTLZ. Vinculin was used as the loading control. E. Immunohistochemistry of PCNA and γH2AX expression in tumor sections, 400x magnification.

Talazoparib, as a PARP inhibitor, induces DNA damage and cell death in BRCA-deficient breast cancer cell lines 35. However, the global regulation of gene expression of Talazoparib vs. NanoTLZ treatment has not been characterized *in vivo*, especially in spontaneous BRCA-deficient mammary gland tumors. Here, we treated Brca1^Co/Co^;MMTV-Cre;p53^+/-^ mice with tumors 4 mm in diameter with 5 doses of i.v. Talazoparib or NanoTLZ (0.33 mg/kg/dose, 3 times a week). Total RNA was extracted from tumors and processed for RNAseq analysis. After five doses of treatment, i.v. Talazoparib significantly (padj<0.05) upregulated 39 genes and downregulated 41 genes. Cluster analysis indicated a similar expression pattern of NanoTLZ compared to Talazoparib (Figure 5A). However, more genes were significantly (padj<0.05) regulated by NanoTLZ: 70 genes were upregulated and 78 genes were downregulated. The Venn diagram summarizes the number of differentially expressed genes compared to the saline group in each treatment group and the overlapping genes found between the comparisons (Figure 5B). The list of differentially expressed genes in NanoTLZ group and i.v. Talazoparib group compared to saline control is summarized in Tables S2 and S3, respectively. Raw data and processed data of the RNAseq were deposited on Gene Expression Omnibus (GEO, GSE125206).

Poly-ADP-ribosylation, a protein post-translational modification, regulates a variety of cellular processes including restarting stalled replication forks, DNA repair, transcription, mitosis and initiating a unique cell death pathway 36. Not surprisingly, many genes involved in transcription, translation, cell cycle and cell death were differentially expressed in treatment groups compared to the saline control (Table S2 and Table S3). *Ngfr* (Nerve growth factor receptor), a known tumor suppressor inhibiting cell growth in breast cancer 37, was upregulated by both NanoTLZ and i.v. Talazoparib. *Bcl11a* (B cell CLL/lymphoma 11A) is overexpressed in triple-negative breast cancer and plays critical roles in stem and progenitor cells 38; it was downregulated by i.v. Talazoparib and NanoTLZ. Oncogenes, like *Kit* and *Myb*, were also downregulated after treatment with i.v. Talazoparib or NanoTLZ, respectively. Downregulation of *Eef1a2* (eukaryotic translation elongation factor 1 alpha 2) and *Top2a* (topoisomerase II alpha) is consistent with growth arrest after treatment in both the i.v. Talazoparib and NanoTLZ groups. Interestingly, a group of myosin related genes were also regulated by Talazoparib treatment, including *Myl1, Myh1, Myh2, Myh4, Mybpc2* and *Mylpf*. Myosins play critical roles in various processes during tumor development, including cell adhesion, migration, and suppression of apoptosis. Accumulating studies suggest that many kinds of myosins are involved in the formation and development of cancer 39-41. In addition, some genes that respond to the DNA damage were upregulated in the treatment groups, such as *Gadd45* and *Sod3*.

Notably, NanoTLZ regulated several immune-related genes including cytokines (e.g. *Cxcl12*, *IL13ra2*) and immunoglobulins (e.g. *Igha, Igkc, Ighg2b, Igj*), which were not affected by i.v. free Talazoparib. Cxcl12 enhances anti-cancer immunity and thus blocks both metastasis and primary tumor growth particularly in breast cancer 42. IL13ra2 (Interleukin-13 receptor α2 chain) has also been shown to inhibit tumorigenicity of breast and pancreatic cancer in animal models 43.

A panel of genes was further validated by real-time PCR using RNA aliquots from the same samples as the RNAseq analysis and additional samples from other mice in each group (N=5 mice, Figure 5C). The PCR results confirmed the RNAseq analysis for *TNFRSF19, Eef1a2, Kit, Top2a, Sod3, and Cxcl12.* Although tumor samples treated with empty nanoparticles were not included in the original RNAseq comparison, they were analyzed using real-time PCR to evaluate the expression of a few genes that were significantly regulated by NanoTLZ. Interestingly, empty nanoparticles significantly (p<0.05) upregulated the expression of *Cxcl12,* suggesting an immune response was induced by the nanocarrier (Figure S5). However, as expected, genes like *Top2a* were not regulated by empty nanoparticles (Figure S5). Because regulation of proliferation, cell cycle arrest, and DNA damage were observed at the level of gene expression, protein expression of biomarkers in these cellular processes including PCNA, CyclinD1, CyclinE1, c-caspase 3, and γH2AX was analyzed by either western blotting or immunohistochemistry (Figure 5D-E). PCNA, a biomarker of proliferation, was significantly (p<0.05) downregulated with NanoTLZ treatment (quantified in Figure S6). There was a striking decrease of Cyclin D1 and Cyclin E1 protein expression in the NanoTLZ treated tumor lysates, indicating cell cycle arrest. γH2AX was detected on tumor sections treated with NanoTLZ (Figure 5E), demonstrating increased DNA damage after treatment. Similar effects on proliferation, cell cycle arrest and DNA damage were also observed in the i.v. TLZ group but at a lower magnitude than the nanoTLZ group (Figure S7A-B).

### NanoTLZ modulates immune cell populations in mammary gland, spleen and tumor

**Figure 6:**
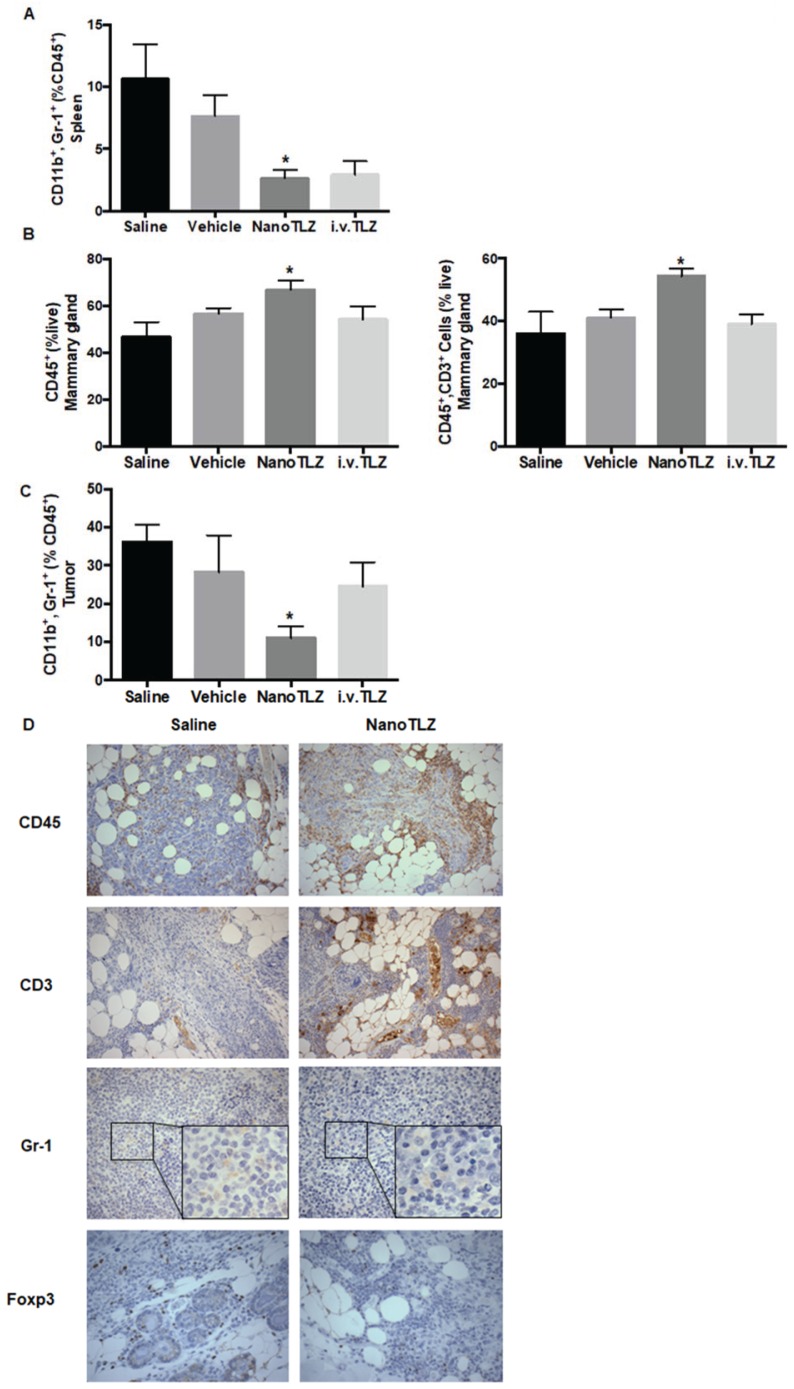
NanoTLZ modulates immune cell populations in BRCA-deficient mice. Brca1^Co/Co^;MMTV-Cre;p53^+/-^ mice bearing tumors (4 mm in diameter) were treated with 5 doses of saline, empty nanoparticle (vehicle), free TLZ (i.v. TLZ) or NanoTLZ, and tumor, spleen and mammary gland without visible tumors were collected for flow cytometry. The percentage of immune populations with significant changes in spleen, mammary gland, or tumor are shown from A to C, respectively. N=5 mice/group. *, p<0.05 vs*.* saline. D. The changes of total immune cells (CD45) and T cells (CD3) in mammary gland were confirmed by immunohistochemistry. The changes in myeloid-derived suppressor cells (MDSCs, Gr-1) in the spleen were also validated using immunohistochemistry, 200x magnification. The change of Foxp3 expression around the tumor was shown by IHC at 400x magnification.

Because NanoTLZ regulated immune-associated genes in the tumor, we then examined the effects of NanoTLZ on immune populations within the tumor microenvironment. Here, we used our Brca1^Co/Co^;MMTV-Cre;p53^+/-^ mouse model and investigated the effects of saline, empty nanoparticle (vehicle), NanoTLZ and i.v. Talazoparib on immune populations. Mice bearing tumors (4 mm in diameter) were treated with either saline, empty nanoparticle, NanoTLZ or i.v. Talazoparib for 5 doses (0.33 mg/kg, 3 times a week). Fresh tumors, spleens and mammary glands without visible tumors were collected and processed for flow cytometry. No significant changes in immune populations were observed in empty nanoparticle treated mice compared to saline control (Figure 6A-C). Both NanoTLZ and i.v. Talazoparib induced a decrease in the percentage of myeloid-derived suppressor cells (MDSCs - CD45^+^, CD11b^+^, Gr-1^+^) in the spleen compared to saline group, but it is only statistically significant (p<0.05) in the NanoTLZ group (Figure 6A). Interestingly, NanoTLZ significantly (p<0.05) increased the total immune cells (CD45^+^) and total T cells (CD45^+^, CD3^+^) in the mammary gland (Figure 6A). These changes were not observed with free i.v. Talazoparib treatment. Importantly, NanoTLZ also induced a striking and significant (p<0.05) decrease of MDSCs, a critical immune-suppressive population, in the tumors, while the change was not significant in the free Talazoparib group (Figure 6C). The results detected by flow cytometry were confirmed by immunohistochemistry (Figure 6D). Although no significant difference was observed in the percentage of CD4^+^ or CD8^+^ T cells in either the NanoTLZ or i.v. Talazoparib group in tumors, the percentage of Foxp3 positive cells around the tumor was significantly (p<0.01) decreased in the NanoTLZ group compared to saline controls (Figure 6D and quantified in Figure S8). The differences in CD45^+^ cells and T cells in the mammary glands were observed at pre-lesion sites where no palpable lesions were detected.

## Discussion

To overcome the limitations of oral administration and increase the specific targeting of tumors, we encapsulated Talazoparib into nano liposomes. Our studies demonstrated that this nano-formulation enhanced the efficacy of Talazoparib with prolonged progression free survival and overall survival in BRCA-deficient mice. Compared to free Talazoparib, NanoTLZ was more effective with higher percentages of tumor regression and complete regression achieved, leading to greater overall survival and longer time to disease progression. Moreover, our nano-formulation also reduced the toxicity of Talazoparib.

Although Talazoparib is a more potent drug than Olaparib and thus administered at a lower dose, it is also more toxic clinically. 55% of patients receiving Talazoparib developed adverse grade 3-4 hematological events, mainly anemia, and 25% of patients treated with this PARP inhibitor presented with alopecia. Other common (≥20%) adverse reactions with Talazoparib treatment in patients include fatigue, decreased appetite, vomiting, and nausea. Our BRCA-deficient mouse model mimics the toxicity observed in human patients after Talazoparib treatment. There was a significant (p<0.05) loss of body weight in the free Talazoparib treatment groups, and alopecia also was observed in both free Talazoparib groups. NanoTLZ, excitingly, had decreased toxicity, evident by the absence of weight loss and alopecia. In addition, worse toxicity has also been observed in clinical trials that combine PARP inhibitors with DNA damaging agents 44-50. Severe side effects experienced during trials with PARP inhibitors and a number of chemotherapeutics required the dose and exposure of PARP inhibitors to be reduced, leading to subtherapeutic doses and ineffective combinations. A clinical trial of oral TLZ in combination with carboplatin required all patients to undergo treatment delay and dose reduction after cycle one due to adverse toxicity 50. With our new nano-liposome formulation, NanoTLZ, which was better tolerated than free TLZ, significantly improved both PFS and OS compared to free TLZ. Future studies will investigate if similar efficacy and toxicity results are observed when NanoTLZ is combined with chemotherapy.

Because of the EPR effect, nano-carriers accumulated in the tumor of the BRCA-deficient mice and greatly improved the specificity of targeting to the tumor. Liposome-PEG nanocarriers also facilitate cellular uptake. In addition, NanoTLZ induced a striking decrease of MDSCs in the tumor, which was not found with free Talazoparib treatment. NanoTLZ also increased the infiltration of T cells into the pre-lesion sites in the mammary gland of BRCA-deficient mice. Although no significant differences in drug levels were detected in tumor lysates 24 hours after treatment with NanoTLZ and i.v. TLZ, the multiple different effects that we describe when comparing NanoTLZ to free Talazoparib are important benefits of this novel delivery system. Although the EPR effect is model-dependent, the improvements to Talazoparib achieved by using a nanoparticle formulation could bring better therapeutic outcomes and improved quality of life during treatment for breast cancer patients if similar EPR effects are observed in humans.

With the advance of nanotechnology and its unique properties, nanomedicine has emerged as a promising approach in cancer therapeutics. Doxil (liposomal formulation of doxorubicin) and Abraxane (albumin-bound nanoparticle of paclitaxel) are two successful examples of drug delivery nanoparticles. Doxil, as the first FDA-approved nano-drug, has a prolonged circulation time with better effects than other forms of doxorubicin hydrochloride and fewer side effects 51. Abraxane, nab-paclitaxel, was designed to overcome the barrier of effective drug delivery with the conventional formulation because of the lipophilic nature of taxane. To date, Abraxane has been approved for the treatment of metastatic breast cancer, non-small cell lung cancer, and pancreatic cancer. By choosing liposomes as the nano-carrier, our study is clinically relevant. Liposomes were the first approved drug-delivery vehicles because of their biocompatibility and biodegradability. Over ten liposomal formulations have been approved for different indications, and numerous other liposomes are being tested in clinical trials 22. Liposomal-based drugs have a series of advantages including greater solubility, increased half-life, the ability to overcome drug resistance, and increased specificity to the target site. Although a drawback of liposomes is a rapid capture and clearance by the RES, it can be overcome by PEGylation of the liposome, which induces steric hindrance.

Several other groups have made nanoformulated PARP inhibitors. A lipid-based injectable nanoformulation of Olaparib was developed to sensitize PTEN/TP53-deficient prostate cancer to radiation 52. Tumor specific proteins can also be conjugated onto liposomes to enhance targeted drug delivery. For example, plectin-targeted liposomes enhance the therapeutic efficacy of a PARP inhibitor (AZ7379) in the treatment of ovarian cancer in animal models 53. Recently, a solid lipid nanoparticle formulation of Talazoparib was reported and has been tested in *BRCA1* mutant triple negative breast cancer cell lines *in vitro*. The solid lipid formulated Talazoparib was more effective than Talazoparib in inhibiting cell growth in breast cancer cell lines and overcame HR-mediated resistance in TNBC cells 54 but was not tested *in vivo*. Here, we developed a novel liposome-based nanoformulation for Talazoparib and, to our knowledge, were the first to extensively evaluate its effects in a spontaneous and clinically relevant mouse model of breast cancer.

Notably, we also investigated the immunomodulatory effects of Talazoparib and compared it to NanoTLZ in this study. In a mouse model of ovarian cancer, Talazoparib increased peritoneal CD8^+^ T cells and NK cells 55. We, for the first time, have characterized immune populations within the tumor microenvironment in a BRCA-deficient breast cancer model. Brca1^Co/Co^;MMTV-Cre;p53^+/-^ mice develop tumors at an average age of 24-32 weeks and recapitulate BRCA-deficient breast cancer in humans. By using this spontaneous tumor model, an intact immune system is present, which allows us to study the endogenous tumor microenvironment. Notably, our data showed that Talazoparib decreased the percentage of MDSCs, which is an immuno-suppressive population. NanoTLZ was more effective than free Talazoparib in reducing MDSCs in the tumor. MDSCs are known to inhibit the host immune response in breast cancer patients and limit the effectiveness of immunotherapies 56. Meanwhile, Foxp3 expression, a marker of T regulatory cells, another immunosuppressive population, was decreased with NanoTLZ treatment. NanoTLZ also had additional effects beyond Talazoparib of modulating immune cells at the pre-lesion sites (Figure 6), suggesting a potential effect on newly developing tumors. RNAseq analysis (Figure 5) suggested that NanoTLZ, rather than Talazoparib, regulated immune-associated genes, which may explain the difference between NanoTLZ and Talazoparib on immune modulation. Interestingly, some effects on immune regulation may be induced by the nano-carriers, as empty nanoparticles upregulated *Cxcl12* expression. Additionally, our preliminary data suggests an increased activation of dendritic cells with NanoTLZ treatment compared to free TLZ, which will be fully explored in future studies.

In addition to the immunomodulation of NanoTLZ, PARP inhibitors have recently been shown to upregulate PD-L1 expression 57. Therefore, there is a strong rationale for combining NanoTLZ with immunotherapies, such as PD1/PDL-1 immune checkpoint inhibitors. With its reduced toxicity, NanoTLZ is also a better compound for combining with other anti-cancer agents. In fact, the first positive results of a clinical trial in breast cancer patients using the combination of immunotherapy with nanomedicine was recently reported. Median progression-free survival was significantly (p<0.05) extended from 5.5 months in patients with metastatic TNBC treated with placebo plus nab-paclitaxel to 7.2 months in patients treated with atezolizumab (anti-PD-L1) plus nab-paclitaxel 58. In the future, we will explore the combination of NanoTLZ and immunotherapy (anti-PD1/PDL-1 antibodies) for treating BRCA-deficient breast cancer.

## Supplementary Material

Supplementary figures and tables.

## Abbreviations

ATM: Ataxia-Telangiesctasia mutated gene
ATR: Ataxia telangiectasia and Rad3 related
BRCA: breast cancer-associated gene
CHK1/2: checkpoint kinase 1/2
Cxcl12: C-X-C motif chemokine 12
Eef1a2: eukaryotic translation elongation factor 1 alpha 2
EPR: enhanced permeability and retention
FANCA: FA Complementation Group A
FANCD2: FA Complementation Group D2
Foxp3: forkhead box P3
HR: homologous recombination
IL13ra2: Interleukin-13 receptor subunit alpha-2
MDSC: myeloid-derived suppressor cell
Mybpc2: Myosin binding protein C
Myh1: striated muscle myosin heavy chain 1
Myl1: Myosin light chain 3
Mylpf: Myosin light chain, phosphorylatable, fast skeletal muscle
NK cells: natural killer cells
PARP: poly (ADP-ribose) polymerase
PEG: polyethylene glycol
PTEN: Phosphatase and tensin homolog
RES: reticuloendothelial system
RIN: RNA integrity number
Sod3: superoxide dismutase 3
Top2a: topoisomerase II alpha
TNBC: triple negative breast cancer
TNFRSF19: Tumor necrosis factor receptor superfamily, member 19
