# Supplementary material for: A nano-liposome formulation of the PARP inhibitor Talazoparib enhances treatment efficacy and modulates immune cell populations in mammary tumors of BRCA-deficient mice
Source: Theranostics. 2019 Aug 14;9(21):6224–38. doi: 10.7150/thno.36281 (PMC6735511; doi:10.7150/thno.36281)
Supplement: Supplementary file 1 — Supplementary figures and tables. [file thnov09p6224s1.pdf]

Figure S1 Validation of free TLZ drug removal after encapsulation. The non-encapsulated drug, which is insoluble in aqueous media, was removed via a syringe filter. A. Chromatogram of a 500 ng/mL TLZ standard. B. To test whether free drug can be removed using this syringe filter method, the total amount of drug in 1 ml of formulation was added to 1 ml of PBS and filtered. This solution was analyzed using the same HPLC protocol as the nanoparticles, and no TLZ was detected.

Figure S2 Cell viability after treatment of vehicle. W780 BRCA-deficient cells were seeded into 96 well plates at 1000 cells/well. Cells were exposed to vehicle (empty nanoparticles) or PBS (vehicle in which NanoTLZ is stored) at the volume equivalent of 10 μM NanoTLZ as used in Fig. 1D. One week after seeding, cell viability was ascertained by the MTS assay. Percent cell viability was normalized to control. Data presented as mean ± SD.

Figure S3 Body weight of BRCA-deficient mice during NanoTLZ or TLZ treatment. $Brca1^{Co/Co}$;MMTV-Cre;$p53^{+/-}$ mice bearing tumors 4 mm in diameter were treated with either NanoTLZ by i.v. or free Talazoparib by gavage (0.33 mg/kg, 3 times a week). Mice were weighed each time before treatment. Data presented as mean ± SEM. N=8/group.

Figure S4 Histology of liver tissues after NanoTLZ treatment. $Brca1^{Co/Co}$;MMTV-Cre;$p53^{+/-}$ mice bearing tumors 4 mm in diameter were treated with either saline or NanoTLZ (0.33 mg/kg, 3 times a week). Mice were euthanized when tumors reached 10 mm in diameter and the liver was collected. Liver tissues were fixed in 10% formalin and histology slides were stained with

Haemotoxylin and Eosin. Representative images are shown for 2 mice per group. 400x magnification.

Figure S5 Empty nanoparticles upreguate the expression of Cxcl12. RNA was isolated from tumors of Brca1$^{Co/Co}$;MMTV-Cre;p53$^{+/-}$ mice treated with empty nanoparticles for 5 doses. The expression of genes, including *Top2a, Sod3, Cxcl12*, that were significantly regulated by NanoTLZ were measured and compared to saline treated tumors. *, $p<0.05$ vs. saline. N=5 mice/group. Data presented as mean ± SEM.

Figure S6 Quantification of western blotting for Fig. 5D. Immunoblots in Fig. 5D were quantified by Image J. Protein level was normalized to vinculin loading control and then saline controls. Data presented as mean ± SEM.

Figure S7 i.v. TLZ decreases proliferation and induces DNA damage in BRCA-deficient tumors. A. Protein expression of PCNA, CyclinD1, CyclinE1 in tumors from Brca1$^{Co/Co}$;MMTV-Cre;p53$^{+/-}$ mice treated with saline or i.v. TLZ. Vinculin was used as the loading control. B. Immunohistochemistry of PCNA and γH2AX in tumor sections. 400x magnification.

Figure S8 Quantification of Foxp3 staining around tumors. IHC staining in Fig. 6D was quantified by Image J. Percentage of Foxp3 positive cells around tumors was calculated. Data presented as mean ± SEM. **, $p<0.01$ vs. saline. N=8/group.

Figure S1

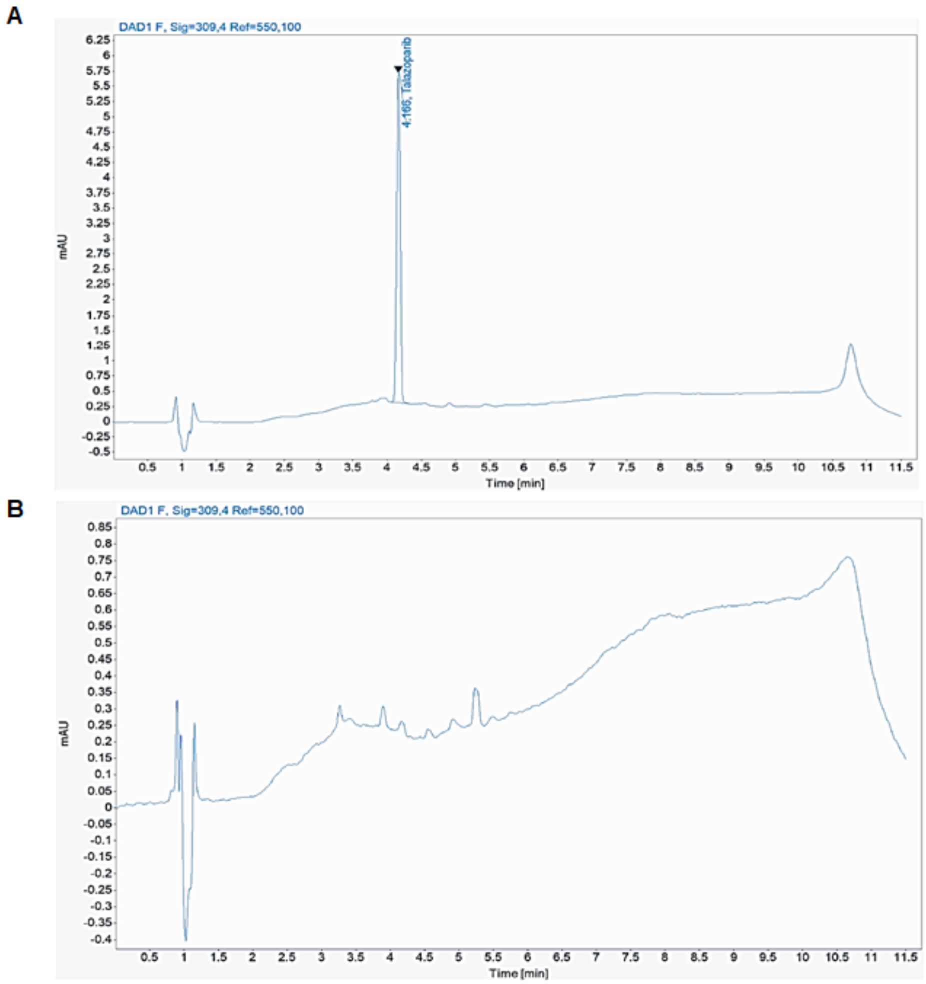

Figure S2

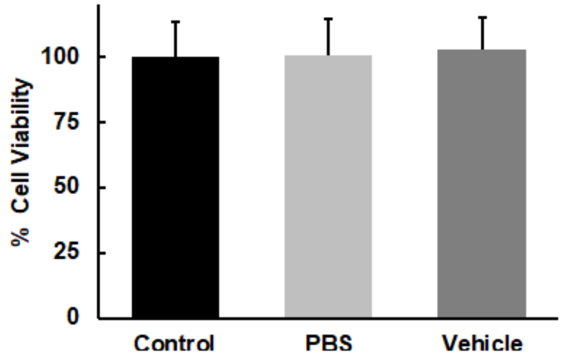

Figure S3

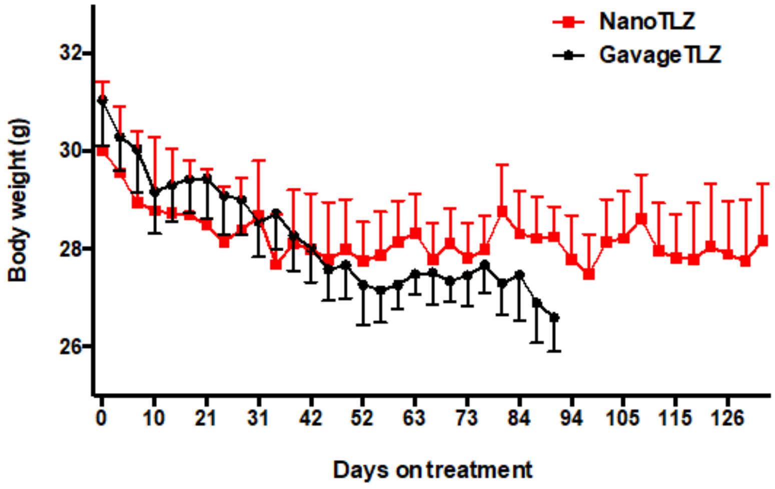

Figure S4

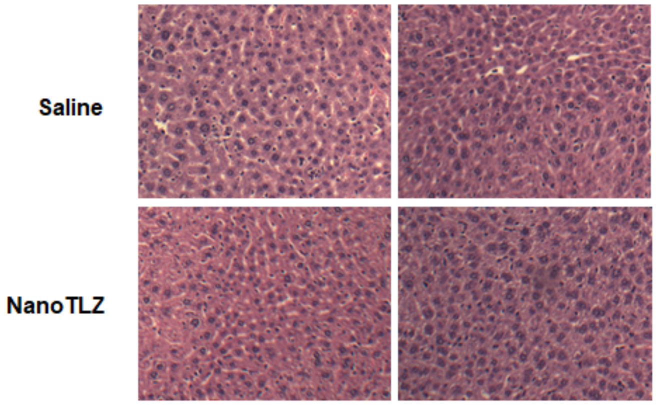

Figure S5

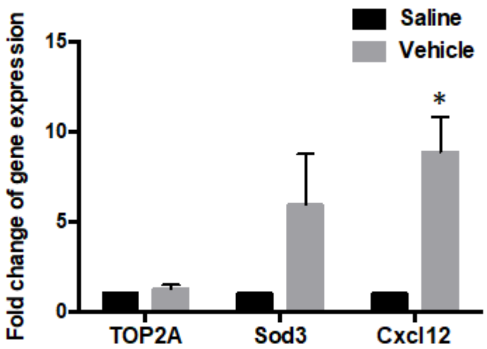

Figure S6

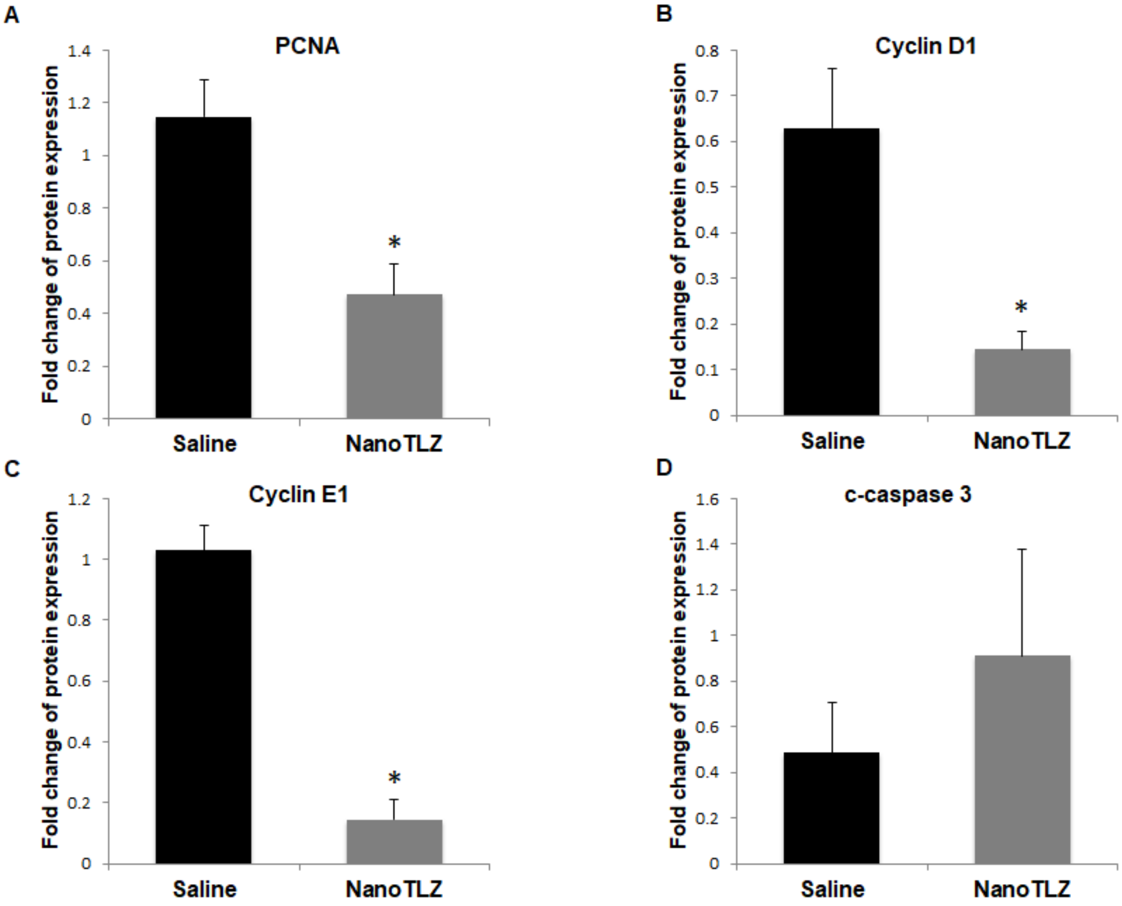

Figure S7

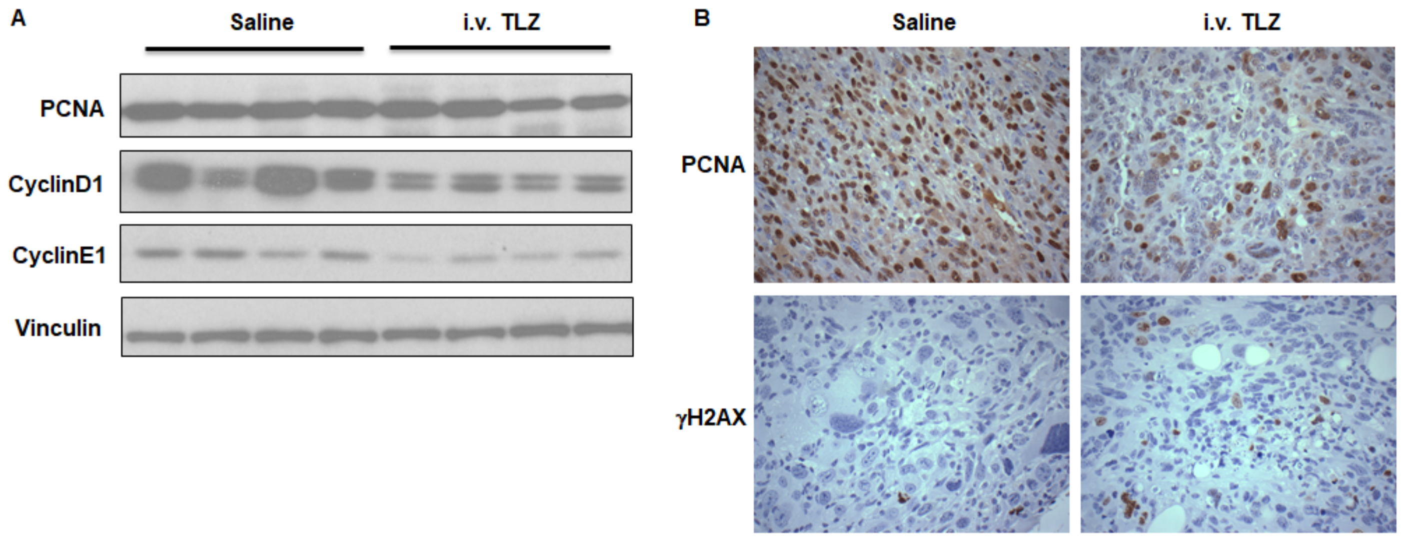

Figure S8

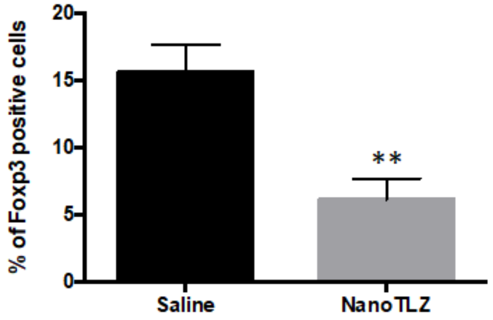

Table S1. Primer sequence

Gene	Forward	Reverse
Kit	TCAACGACCTTCCCGAAGGCACCA	CTGGTGGTTCAGAGTTCCATAGAC
Top2a	TTCTTGATATGCCCCTTTGG	GCTTCAACAGCCTCCAATTC
Sod3	GTGTCCCAAGACAATC	GTGCTATGGGGACAGG
Tnfrsf19	CTGGATTTGAAGTTTGTCTG	CGTGTTTATTCCTGCTACTC
Eef1a2	GCCACGATCAGCACTGCG	CAAGCGGACCATCGAGAAGT
Myb	TGTCCTCAAAGCCTTTACCG	TTCACGTATTTCCGAGCCG
GAPDH	AGGTCGGTGTGAACGGATTTG	TGTAGACCATGTAGTTGAGGTCA

Table S2. Differentially expressed genes in the tumor between saline and i.v.TLZ groups

Gene_ID	Saline	i.v. TLZ	log2Fold Change	pvalue	padj	GeneName	Chrom	GeneDescription
ENSMUSG00000000120	29.23307547	258.6173387	2.3279	6.04E-05	0.019301	Ngfr	chr11	nerve_growth_factor_receptor_(TNFR_superfamily_member_16)
ENSMUSG00000000861	1014.639891	122.3377625	-2.212	0.0001863	0.040763	Bcl11a	chr11	B_cell_CLL/lymphoma_11A_(zinc_finger_protein)
ENSMUSG00000004347	206.8500299	9.805225252	-2.5869	6.47E-05	0.019579	Pde1c	chr6	phosphodiesterase_1C
ENSMUSG00000004360	72.00858758	1.734986211	-2.6401	6.58E-05	0.019579	9330159F19Rik	chr10	RIKEN_cDNA_9330159F19_gene
ENSMUSG00000005672	6618.945697	2182.89878	-1.4499	0.00019519	0.041239	Kit	chr5	kit_oncogene
ENSMUSG00000005716	230.4925517	0.717262265	-4.6561	7.54E-14	3.25E-10	Pvalb	chr15	parvalbumin
ENSMUSG00000006457	365.2921968	4.886816862	-3.6079	1.73E-08	3.95E-05	Actn3	chr19	actinin_alpha_3
ENSMUSG00000009394	17.15143524	195.3436831	2.4552	4.90E-05	0.017091	Syn2	chr6	synapsin_II
ENSMUSG00000015519	305.4260516	5.160700621	-3.0007	5.13E-06	0.0035771	2310057J18Rik	chr10	RIKEN_cDNA_2310057J18_gene
ENSMUSG00000016349	77.2503192	1.075893397	-2.502	0.00017523	0.039824	Eef1a2	chr2	eukaryotic_translation_elongation_factor_1_alpha_2
ENSMUSG00000018102	391.9271153	1543.116925	1.7394	6.13E-05	0.019301	Hist1h2bc	chr13	histone_cluster_1_H2bc
ENSMUSG00000018907	170.4773238	1327.929647	2.1702	0.0002169	0.04465	Alox12e	chr11	arachidonate_lipoxygenase_epidermal
ENSMUSG00	55.38212112	0.313844758	-2.9882	6.57E-06	0.0040586	Trdn	chr10	triadin

000019787								
ENSMUSG00 000020334	29.11473953	206.9442724	2.4047	3.86E-07	0.0004765 5	Slc22a4	chr11	solute_carrier_family_22_(organic_cation_transporter)_member_4
ENSMUSG00 000020914	6468.651913	1717.911913	-1.7216	1.66E-05	0.0074244	Top2a	chr11	topoisomerase_(DNA)_II_alpha
ENSMUSG00 000021070	15.17087595	90.22290885	2.0271	0.000188	0.040763	Bdkrb2	chr12	bradykinin_receptor_beta_2
ENSMUSG00 000022483	290.3343326	3291.191384	3.115	8.10E-14	3.25E-10	Col2a1	chr15	collagen_type_II_alpha_1
ENSMUSG00 000022543	119.8691234	18.29671379	-2.1553	5.59E-05	0.018708	4930451G0 9Rik	chr16	RIKEN_cDNA_4930451G09 _gene
ENSMUSG00 000023484	5.481498369	58.33632045	2.2859	0.000227 36	0.046212	Prph	chr15	peripherin
ENSMUSG00 000024365	8.652294956	168.8876653	2.8428	4.67E-06	0.0035698	Cyp21a1	chr17	cytochrome_P450_family_21_subfamily_a_polypeptide_1
ENSMUSG00 000024471	47.11432513	0	-2.9441	9.73E-06	0.0051501	Myot	chr18	myotilin
ENSMUSG00 000025347	1.839590732	45.2392207	2.5699	7.93E-05	0.021695	Mettl7b	chr10	methyltransferase_like_7B
ENSMUSG00 000025875	333.093697	1415.539776	1.8298	3.02E-05	0.012117	Tspan17	chr13	tetraspanin_17
ENSMUSG00 000025930	10.91563416	99.5420372	2.4863	5.35E-06	0.0035771	Msc	chr1	musculin
ENSMUSG00 000026258	3608.091728	234.882091	-2.5095	7.62E-05	0.021455	3110079O1 5Rik	chr1	RIKEN_cDNA_3110079O15 _gene
ENSMUSG00 000026586	246.9122526	800.4488124	1.5339	9.47E-05	0.024522	Prrx1	chr1	paired_related_homeobox_1
ENSMUSG00	9.760283266	101.3868182	2.2964	0.000190	0.040763	Tnn	chr1	tenascin_N

000026725				4				
ENSMUSG00 000027463	77.04624868	314.1877113	1.7462	0.000150 6	0.035561	Slc52a3	chr2	solute_carrier_protein_famil y_52_member_3
ENSMUSG00 000027559	414.4916034	10.0603722	-3.2926	2.16E-07	0.0002888 9	Car3	chr3	carbonic_anhydrase_3
ENSMUSG00 000030022	1936.508135	532.8993384	-1.6589	6.48E-05	0.019579	Adamts9	chr6	a_disintegrin-like_and_metal lopeptidase_(reprolysin_typ e)_with_thrombospondin_ty pe_1_motif_9
ENSMUSG00 000030110	16.81106862	91.39934081	1.9615	0.000199 07	0.041513	Ret	chr6	ret_proto-oncogene
ENSMUSG00 000030200	928.6676251	105.0267708	-2.4657	6.24E-06	0.0040084	Bcl2l14	chr6	BCL2-like_14_(apoptosis_fa cilitator)
ENSMUSG00 000030222	431.1318791	88.53535083	-1.943	4.21E-05	0.015178	Rerg	chr6	RAS-like_estrogen-regulate d_growth-inhibitor
ENSMUSG00 000030399	260.6974218	2.125020642	-3.9834	3.51E-10	9.40E-07	Ckm	chr7	creatine_kinase_muscle
ENSMUSG00 000030510	121.560908	18.41376929	-2.2381	1.03E-05	0.0051501	Cers3	chr7	ceramide_synthase_3
ENSMUSG00 000030672	473.1342088	18.13061124	-3.0574	1.00E-06	0.0009457 5	Mylpf	chr7	myosin_light_chain_phosph orylatable_fast_skeletal_mu scle
ENSMUSG00 000030730	633.0854991	18.64109335	-3.2876	1.42E-07	0.0002075 6	Atp2a1	chr7	ATPase_Ca++_transporting _cardiac_muscle_fast_twitc h_1
ENSMUSG00 000031626	3890.371794	1100.720875	-1.6389	4.25E-05	0.015178	Sorbs2	chr8	sorbin_and_SH3_domain_c ontaining_2
ENSMUSG00 000031972	1956.227592	5.9452184	-4.5047	1.71E-12	5.50E-09	Acta1	chr8	actin_alpha_1_skeletal_mus cle
ENSMUSG00	43.51294258	264.5311495	2.026	0.000246	0.049549	Ptgs2	chr1	prostaglandin-endoperoxide

000032487				86				_synthase_2
ENSMUSG00 000033196	96.04742938	0.345248056	-2.9314	1.07E-05	0.0051905	Myh2	chr11	myosin_heavy_polypeptide_ 2_skeletal_muscle_adult
ENSMUSG00 000036139	11.60877588	125.5014975	2.5452	1.01E-05	0.0051501	Hoxc9	chr15	homeobox_C9
ENSMUSG00 000037362	26.2393884	218.6582485	2.34	3.13E-05	0.012258	Nov	chr15	nephroblastoma_overexpres sed_gene
ENSMUSG00 000037960	481.0879019	1627.809767	1.5634	0.000188 01	0.040763	1110007C0 9Rik	chr13	RIKEN_cDNA_1110007C09 _gene
ENSMUSG00 000038670	469.7441164	36.65250186	-2.9114	6.13E-08	9.84E-05	Mybpc2	chr7	myosin_binding_protein_C_f ast-type
ENSMUSG00 000039137	124.2125776	1040.412652	2.5997	5.26E-08	9.38E-05	Whrn	chr4	whirlin
ENSMUSG00 000042156	192.2499112	49.98051525	-1.6952	0.000131 57	0.033009	Dzip1	chr14	DAZ_interacting_protein_1
ENSMUSG00 000043613	76.13595914	1264.529018	2.9152	8.70E-07	0.0009315 7	Mmp3	chr9	matrix_metallopeptidase_3
ENSMUSG00 000044155	1052.36644	14380.93829	2.4371	0.000113 15	0.028839	Lsm8	chr6	LSM8_homolog_U6_small_ nuclear_RNA_associated_(S._cerevisiae)
ENSMUSG00 000044349	1904.015011	78.04641385	-3.061	7.91E-07	0.0009075 8	Snhg11	chr2	small_nucleolar_RNA_host_ gene_11
ENSMUSG00 000045532	10.04215269	62.55217387	2.1515	2.84E-05	0.011703	C1ql1	chr11	complement_component_1_ q_subcomponent-like_1
ENSMUSG00 000049134	146.9349053	6.227319725	-2.905	3.92E-06	0.003146	Nrap	chr19	nebulin-related_anchoring_p rotein
ENSMUSG00 000049723	224.0177778	2771.080596	2.7619	9.87E-07	0.0009457 5	Mmp12	chr9	matrix_metallopeptidase_12
ENSMUSG00 000050578	263.0220625	3088.69546	2.372	0.000139 03	0.03332	Mmp13	chr9	matrix_metallopeptidase_13

ENSMUSG00 000052135	102.4260603	761.3440225	2.3192	1.20E-05	0.0056792	Foxo6	chr4	forkhead_box_O6
ENSMUSG00 000054753	58.9555992	402.4078268	2.2273	2.44E-05	0.010424	AU018091	chr7	expressed_sequence_AU01 8091
ENSMUSG00 000056328	192.907765	5.680797164	-2.6982	4.08E-05	0.015178	Myh1	chr11	myosin_heavy_polypeptide_ 1_skeletal_muscle_adult
ENSMUSG00 000056895	0.602267229	44.26661411	3.1516	1.23E-06	0.0010373	Hist3h2ba	chr11	histone_cluster_3_H2ba
ENSMUSG00 000057003	1697.048389	2.053468115	-4.9215	9.41E-15	9.46E-11	Myh4	chr11	myosin_heavy_polypeptide_ 4_skeletal_muscle
ENSMUSG00 000057967	0.377572761	24.56331425	2.6816	5.10E-05	0.01743	Fgf18	chr11	fibroblast_growth_factor_18
ENSMUSG00 000058740	0	40.29814579	2.9471	9.45E-06	0.0051501	Kcnt1	chr2	potassium_channel_subfami ly_T_member_1
ENSMUSG00 000059412	12.48275609	174.703999	2.4763	7.97E-05	0.021695	Fxyd2	chr9	FXYD_domain-containing_io n_transport_regulator_2
ENSMUSG00 000060548	420.1201105	74.29550326	-2.0415	7.27E-05	0.021093	Tnfrsf19	chr14	tumor_necrosis_factor_rece ptor_superfamily_member_1 9
ENSMUSG00 000060981	42.2553062	308.2381984	2.3984	1.14E-06	0.0010168	Hist1h4h	chr13	histone_cluster_1_H4h
ENSMUSG00 000061462	104.5190405	7.09730147	-2.497	7.36E-05	0.021093	Obscn	chr11	obscurin_cytoskeletal_calm odulin_and_titin-interacting_ RhoGEF
ENSMUSG00 000061718	453.7611861	96.12195317	-1.8652	0.000168 2	0.039143	Ppp1r1b	chr11	protein_phosphatase_1_reg ulatory_(inhibitor)_subunit_1 B
ENSMUSG00 000061723	721.6822142	17.51234792	-3.4896	1.97E-08	3.95E-05	Tnnt3	chr7	troponin_T3_skeletal_fast
ENSMUSG00	307.6309278	0.313844758	-4.855	1.18E-14	9.46E-11	Myl1	chr1	myosin_light_polypeptide_1

000061816								
ENSMUSG00000064325	0.292936353	66.34815634	2.683	5.74E-05	0.018817	Hhip	chr8	Hedgehog-interacting_protein
ENSMUSG00000068697	57.51856656	3.084575137	-2.6619	2.47E-05	0.010424	Myoz1	chr14	myozenin_1
ENSMUSG00000069267	2.151585404	35.12836826	2.4372	0.00013756	0.03332	Hist1h3b	chr13	histone_cluster_1_H3b
ENSMUSG00000069793	428.8851915	102.0452216	-1.8509	6.83E-06	0.0040604	Slfn9	chr11	schlafen_9
ENSMUSG00000070469	6.529580245	82.36781028	2.4348	8.92E-05	0.023865	Adamtsl3	chr7	ADAMTS-like_3
ENSMUSG00000072941	133.8028669	1714.567047	2.6342	9.86E-06	0.0051501	Sod3	chr5	superoxide_dismutase_3_extracellular
ENSMUSG00000079092	21.61231318	756.7934819	2.9544	5.18E-06	0.0035771	Prl2c2	chr13	prolactin_family_2_subfamily_c_member_2
ENSMUSG00000084141	16.18133994	122.5912608	2.2149	9.41E-05	0.024522	Olfr1372-ps1	chr11	olfactory_receptor_1372_pseudogene_1
ENSMUSG00000085440	27.19085645	2.394079024	-2.3439	0.00013779	0.03332	Sorbs2os	chr8	sorbin_and_SH3_domain_containing_2_opposite_strand
ENSMUSG00000087075	162.7810845	5.160172257	-2.8303	1.32E-05	0.0060529	A230065H16Rik	chr12	RIKEN_cDNA_A230065H16_gene
ENSMUSG00000090125	5.414166977	68.16322815	2.5146	3.51E-05	0.01341	Pou3f1	chr4	POU_domain_class_3_transcription_factor_1
ENSMUSG00000096847	4.414358175	44.29455257	2.2901	0.00017609	0.039824	Tmem151b	chr17	transmembrane_protein_151B

Table S3. Differentially expressed genes in the tumor between saline and NanoTLZ groups

Gene_ID	Saline	NanoTLZ	log2Fold Change	pvalue	padj	GeneName	Chrom	GeneDescription
ENSMUSG00000000120	29.45828816	532.0126907	3.2297	2.44E-10	6.51E-07	Ngfr	chr11	nerve_growth_factor_receptor_(TNFR_superfamily_member_16)
ENSMUSG00000000861	1026.029208	127.3403145	-2.1255	0.00014293	0.023416	Bcl11a	chr11	B_cell_CLL/lymphoma_11A_(zinc_finger_protein)
ENSMUSG00000001281	44.65153949	142.0003496	1.4818	0.00012875	0.022469	Itgb7	chr15	integrin_beta_7
ENSMUSG00000002105	498.0506683	1367.228011	1.3191	0.00026496	0.035157	Slc39a13	chr2	solute_carrier_family_39_(metal_ion_transporter)_member_13
ENSMUSG00000004360	72.81831316	1.273701469	-2.2062	0.00035005	0.041022	9330159F19Rik	chr10	RIKEN_cDNA_9330159F19_gene
ENSMUSG00000005716	232.9190197	0	-4.7377	6.38E-16	5.12E-12	Pvalb	chr15	parvalbumin
ENSMUSG00000006398	1774.195258	750.2606927	-1.1529	0.000292	0.037504	Cdc20	chr4	cell_division_cycle_20
ENSMUSG00000006457	369.0773943	3.06205181	-4.1398	1.03E-12	3.31E-09	Actn3	chr19	actinin_alpha_3
ENSMUSG00000007908	261.7911664	19.8122058	-2.5038	1.23E-05	0.0041056	Hmgcll1	chr9	3-hydroxymethyl-3-methylglutaryl-Coenzyme_A_lyase-like_1
ENSMUSG0	17.24660109	187.0411487	2.2715	8.65E-05	0.017355	Syn2	chr6	synapsin_II

0000009394								
ENSMUSG00000012889	63.39416677	385.7726211	1.9665	0.00019842	0.02896	Podnl1	chr8	podocan-like_1
ENSMUSG00000013338	140.4695728	19.68673594	-2.0038	0.00033294	0.039595	Fer1l4	chr2	fer-1-like_4_(C._elegans)
ENSMUSG00000015519	307.0001139	6.146186378	-2.8743	2.48E-06	0.0012049	2310057J18Rik	chr10	RIKEN_cDNA_2310057J18_gene
ENSMUSG00000015709	570.9078436	109.6499561	-1.8504	0.00030116	0.037775	Arnt2	chr7	aryl_hydrocarbon_receptor_nuclear_translocator_2
ENSMUSG00000015962	63.47800858	288.9538394	1.8364	5.03E-05	0.012271	1700016C15Rik	chr1	RIKEN_cDNA_1700016C15_gene
ENSMUSG00000016349	78.03149939	1.348643718	-2.4637	6.54E-05	0.014583	Eef1a2	chr2	eukaryotic_translation_elongation_factor_1_alpha_2
ENSMUSG00000016984	642.0585875	149.6678937	-1.7243	0.00026474	0.035157	Etaa1	chr11	Ewing_tumor-associated_antigen_1
ENSMUSG00000016995	73.01121415	1245.290423	2.7643	1.29E-06	0.00082735	Matn4	chr2	matrilin_4
ENSMUSG00000017007	2.194507593	36.68254654	2.4787	2.83E-05	0.0082588	Rbpjl	chr2	recombination_signal_binding_protein_for_immunoglobulin_kappa_J_region-like
ENSMUSG00000018102	394.5145984	1702.979655	1.8703	1.83E-06	0.00094765	Hist1h2bc	chr13	histone_cluster_1_H2bc
ENSMUSG00000019787	55.98984409	0	-2.7898	6.20E-06	0.0026881	Trdn	chr10	triadin
ENSMUSG00000019982	331.836127	32.76133855	-2.2817	6.14E-05	0.01408	Myb	chr10	myeloblastosis_oncogene
ENSMUSG0	249.6228823	46.81218999	-1.9025	0.000156	0.02446	Mtfr2	chr10	mitochondrial_fissio

0000019992				92				n_regulator_2
ENSMUSG0 0000019997	1088.249733	3734.301413	1.5349	0.000299 99	0.037775	Ctgf	chr10	connective_tissue_ growth_factor
ENSMUSG0 0000020061	90.22197576	0.318425367	-2.7429	8.86E-06	0.003387	Mybpc1	chr10	myosin_binding_pro tein_C_slow-type
ENSMUSG0 0000020062	4080.592293	840.9078865	-1.8218	0.000221 53	0.030928	Slc5a8	chr10	solute_carrier_famil y_5_(iodide_transp orter)_member_8
ENSMUSG0 0000020128	2388.076619	766.3821696	-1.4381	0.000386 46	0.044005	Vps54	chr11	vacuolar_protein_so rting_54_(yeast)
ENSMUSG0 0000020159	6122.970294	2162.131827	-1.3621	0.000145 27	0.023514	Gabrp	chr11	gamma-aminobutyri c_acid_(GABA)_A_r eceptor_pi
ENSMUSG0 0000020334	29.35235659	98.58870444	1.5338	0.000138 44	0.022915	Slc22a4	chr11	solute_carrier_famil y_22_(organic_catio n_transporter)_me mber_4
ENSMUSG0 0000020362	3962.075123	1683.040403	-1.143	0.000425 36	0.046775	Cnot6	chr11	CCR4-NOT_transcri ption_complex_sub unit_6
ENSMUSG0 0000021453	562.9502783	1206.531702	1.0351	0.000368 18	0.042251	Gadd45g	chr13	growth_arrest_and_ DNA-damage-induci ble_45_gamma
ENSMUSG0 0000022144	20.51237319	170.654413	2.5735	1.16E-08	1.70E-05	Gdnf	chr15	glial_cell_line_deriv ed_neurotrophic_fa ctor
ENSMUSG0 0000022483	292.8153973	11358.47217	4.5123	1.17E-25	1.89E-21	Col2a1	chr15	collagen_type_II_al pha_1
ENSMUSG0 0000022512	1152.366739	416.6179232	-1.327	0.000260 07	0.035157	Cldn1	chr16	claudin_1

ENSMUSG00000022543	120.9908705	25.11770295	-1.8416	0.00013069	0.022562	4930451G09Rik	chr16	RIKEN_cDNA_4930451G09_gene
ENSMUSG00000024365	8.696754759	166.7797645	2.6043	1.15E-05	0.0040676	Cyp21a1	chr17	cytochrome_P450_family_21_subfamily_a_polypeptide_1
ENSMUSG00000024471	47.62694406	0	-2.7276	9.96E-06	0.0037198	Myot	chr18	myotilin
ENSMUSG00000024803	167.395022	892.6299439	1.8852	0.00021314	0.030553	Ankrd1	chr19	ankyrin_repeat_domain_1_(cardiac_muscle)
ENSMUSG00000025347	1.850421602	31.81115614	2.3991	5.70E-05	0.013468	Mettl7b	chr10	methyltransferase_like_7B
ENSMUSG00000025665	319.9564552	10.88944275	-2.4425	6.93E-05	0.014841	Rps6ka6	chrX	ribosomal_protein_S6_kinase_polypeptide_6
ENSMUSG00000025875	335.1941503	1093.408115	1.5127	0.00011128	0.020422	Tspan17	chr13	tetraspanin_17
ENSMUSG00000026308	61.78627977	200.4399705	1.4842	0.00030069	0.037775	Klhl30	chr1	kelch-like_30
ENSMUSG00000026471	424.0871358	133.6658782	-1.4893	9.03E-05	0.017907	Mr1	chr1	major_histocompatibility_complex_class_I-related
ENSMUSG00000026475	644.0500811	3957.081119	1.9196	0.00041743	0.046329	Rgs16	chr1	regulator_of_G-protein_signaling_16
ENSMUSG00000026725	9.816735293	74.11143699	2.248	1.21E-05	0.0041056	Tnn	chr1	tenascin_N
ENSMUSG00000027913	1.79406282	29.57491139	2.1809	0.0003319	0.039595	Crct1	chr3	cysteine-rich_C-terminal_1
ENSMUSG00000027962	86.8273693	489.9251045	2.1196	1.48E-06	0.0008477	Vcam1	chr3	vascular_cell_adhesion_molecule_1

ENSMUSG00000028020	135.5809621	8.226358435	-2.2803	0.00016861	0.025299	Glrb	chr3	glycine_receptor_beta_subunit
ENSMUSG00000028464	2013.633211	745.1549172	-1.2963	0.00036843	0.042251	Tpm2	chr4	tropomyosin_2_beta
ENSMUSG00000028600	24.04628903	97.89666234	1.7407	4.77E-05	0.012185	Podn	chr4	podocan
ENSMUSG00000028678	765.4908167	308.2762706	-1.2274	5.32E-05	0.012742	Kif2c	chr4	kinesin_family_member_2C
ENSMUSG00000029755	1.79406282	58.95269419	2.1848	0.000402	0.045134	Dlx5	chr6	distal-less_homeobox_5
ENSMUSG00000029816	81.33684347	619.1406188	2.4709	3.21E-08	3.68E-05	Gpnmb	chr6	glycoprotein_(transmembrane)_nmb
ENSMUSG00000030200	937.9033169	146.2139676	-2.1505	1.15E-05	0.0040676	Bcl2l14	chr6	BCL2-like_14_(apoptosis_facilitator)
ENSMUSG00000030222	434.9175414	105.30581	-1.7824	1.66E-05	0.005212	Rerg	chr6	RAS-like_estrogen-regulated_growth-inhibitor
ENSMUSG00000030399	263.3920531	0.661106986	-4.2996	3.73E-13	1.50E-09	Ckm	chr7	creatine_kinase_muscle
ENSMUSG00000030607	95.94046441	2939.06666	3.1758	4.58E-08	4.90E-05	Acan	chr7	aggrecan
ENSMUSG00000030672	478.4583843	18.42826445	-2.9206	7.02E-07	0.00053691	Mylpf	chr7	myosin_light_chain_phosphorylatable_fast_skeletal_muscle
ENSMUSG00000030730	639.9107707	15.59338679	-3.5288	6.82E-10	1.22E-06	Atp2a1	chr7	ATPase_Ca++_transporting_cardiac_muscle_fast_twitch_1
ENSMUSG00000030761	180.161366	567.7877463	1.4857	7.36E-05	0.015345	Myo7a	chr7	myosin_VIIA
ENSMUSG0	187.9368911	13.80628934	-2.2785	0.000136	0.022763	Slc6a14	chrX	solute_carrier_famil

0000031089				11				y_6_(neurotransmitter_transporter)_member_14
ENSMUSG00000031289	5.487602326	34.20448597	1.9656	0.00021572	0.030649	Il13ra2	chrX	interleukin_13_receptor_alpha_2
ENSMUSG00000031626	3921.094373	1464.661684	-1.3129	5.95E-05	0.013833	Sorbs2	chr8	sorbin_and_SH3_domain_containing_2
ENSMUSG00000031740	566.3385029	1878.309068	1.4963	0.00038964	0.044054	Mmp2	chr8	matrix_metallopeptidase_2
ENSMUSG00000031841	170.3074118	1004.152138	1.9843	0.00011177	0.020422	Cdh13	chr8	cadherin_13
ENSMUSG00000032358	133.9768513	8.376242933	-2.257	0.00019712	0.02896	Fam83b	chr9	family_with_sequence_similarity_83_member_B
ENSMUSG00000032925	14.89280888	75.18239447	1.8826	0.00011814	0.021312	Itgbl1	chr14	integrin_beta-like_1
ENSMUSG00000033590	171.6278009	17.90926859	-2.174	0.00016232	0.024819	Myo5c	chr9	myosin_VC
ENSMUSG00000034463	884.2046717	283.8636042	-1.4583	0.00016404	0.024847	Scara3	chr14	scavenger_receptor_class_A_member_3
ENSMUSG00000034675	572.9488153	2902.278924	2.0369	8.83E-07	0.00061654	Dbn1	chr13	drebrin_1
ENSMUSG00000035385	110.9264937	555.1662422	1.9202	3.78E-05	0.010293	Ccl2	chr11	chemokine_(C-C_motif)_ligand_2
ENSMUSG00000036523	7.136209348	100.9474226	2.4625	2.46E-05	0.0073201	Greb1	chr12	gene_regulated_by_estrogen_in_breast_cancer_protein
ENSMUSG00000036585	29.89988844	110.1666045	1.631	8.54E-05	0.017354	Fgf1	chr18	fibroblast_growth_factor_1

ENSMUSG00000036832	940.1916061	78.89586243	-2.4691	1.25E-05	0.0041056	Lpar3	chr3	lysophosphatidic_acid_receptor_3
ENSMUSG00000037129	563.4008298	116.3649799	-1.7973	0.0003413	0.040292	Tmprss13	chr9	transmembrane_protease_serine_13
ENSMUSG00000037139	38.75199526	2.403118319	-2.1519	0.00041842	0.046329	Myom3	chr4	myomesin_family_member_3
ENSMUSG00000037610	80.19701859	12.21508311	-2.1036	3.96E-05	0.010607	Kcnmb2	chr3	potassium_large_conductance_calcium-activated_channel_subfamily_M_beta_member_2
ENSMUSG00000037852	2473.241903	11572.39564	1.7843	0.00028308	0.036652	Cpe	chr8	carboxypeptidase_E
ENSMUSG00000038077	16.18865176	179.2384522	2.2958	7.14E-05	0.015092	Kcna6	chr6	potassium_voltage-gated_channel_shaker-related_subfamily_member_6
ENSMUSG00000038239	39.44669237	3.382650672	-2.2931	7.77E-05	0.015994	Hrc	chr7	histidine_rich_calcium_binding_protein
ENSMUSG00000038521	127.5887876	630.4394769	1.9431	1.47E-05	0.0047148	C1s1	chr6	complement_component_1_s_subcomponent_1
ENSMUSG00000038670	474.2706529	39.3228014	-2.8603	6.75E-09	1.08E-05	Mybpc2	chr7	myosin_binding_protein_C_fast-type
ENSMUSG00000038916	79.6416917	1.715581588	-2.3122	0.00018068	0.026859	Soga3	chr10	SOGA_family_member_3
ENSMUSG00000039084	66.10132305	234.6937098	1.6522	6.01E-06	0.0026812	Chad	chr11	chondroadherin
ENSMUSG00000039137	124.9576517	456.3736392	1.5836	0.00036107	0.042007	Whrn	chr4	whirlin

ENSMUSG00000039697	837.1920821	245.800906	-1.6229	1.73E-06	0.00092632	Ncoa7	chr10	nuclear_receptor_coactivator_7
ENSMUSG00000040021	2016.584894	726.6046803	-1.3294	0.00028081	0.036652	Lats1	chr10	large_tumor_suppressor
ENSMUSG00000040649	9.702125571	201.976701	2.3776	9.51E-05	0.018404	Rimklb	chr6	ribosomal_modification_protein_rimK-like_family_member_B
ENSMUSG00000041202	7.945437999	76.7305963	2.2241	9.23E-05	0.018063	Pla2g2d	chr4	phospholipase_A2_group_IID
ENSMUSG00000042099	133.9238465	448.0564936	1.4999	0.00043956	0.048008	Kank3	chr17	KN_motif_and_ankyrin_repeat_domains_3
ENSMUSG00000042357	72.02547953	8.686894926	-2.1447	0.00012143	0.021661	Gjb5	chr4	gap_junction_protein_beta_5
ENSMUSG00000042363	3405.207771	720.1961864	-1.8266	0.00013481	0.022763	Lgalsl	chr11	lectin_galactoside_binding-like
ENSMUSG00000042401	3.126763242	45.14746831	2.4863	1.85E-05	0.0056107	Crtac1	chr19	cartilage_acidic_protein_1
ENSMUSG00000042428	143.2845873	1093.355885	2.443	9.74E-08	9.78E-05	Mgat3	chr15	mannoside_acetylglucosaminyltransferase_3
ENSMUSG00000043613	76.5570707	2125.40235	3.225	1.50E-08	2.01E-05	Mmp3	chr9	matrix_metallopeptidase_3
ENSMUSG00000044006	11.77334934	313.2964969	2.9854	3.35E-07	0.00026871	Cilp2	chr8	cartilage_intermediate_layer_protein_2
ENSMUSG00000044043	21.60932905	97.28095823	1.8844	7.14E-06	0.0029385	Pcdhb14	chr18	protocadherin_beta_14
ENSMUSG00000044155	1061.809099	15689.09807	2.4092	4.86E-05	0.012185	Lsm8	chr6	LSM8_homolog_U6_small_nuclear_RN

								A_associated_(S._cerevisiae)
ENSMUSG00000044338	146.3166592	370.3737678	1.2338	0.00020172	0.029177	Aplnr	chr2	apelin_receptor
ENSMUSG00000044708	57.94101212	294.3848265	1.9076	6.83E-05	0.014815	Kcnj10	chr1	potassium_inwardly-rectifying_channel_subfamily_J_member_10
ENSMUSG00000045699	27.99481751	1.972279579	-2.3033	9.95E-05	0.019023	Gm21411	chr4	predicted_gene_21411
ENSMUSG00000047104	35.75493643	231.1461244	2.0254	0.00013379	0.022763	Pbp2	chr6	phosphatidylethanolamine_binding_protein_2
ENSMUSG00000047420	69.70405608	314.1737529	1.9553	1.20E-07	0.00011312	Fam180a	chr6	family_with_sequence_similarity_180_member_A
ENSMUSG00000047562	33.22097986	710.1543941	2.6766	6.89E-06	0.0029093	Mmp10	chr9	matrix_metallopeptidase_10
ENSMUSG00000049134	148.5864918	5.845322873	-2.847	1.46E-06	0.0008477	Nrap	chr19	nebulin-related_anchoring_protein
ENSMUSG00000049723	225.2702745	2220.417544	2.4477	5.13E-06	0.0023548	Mmp12	chr9	matrix_metallopeptidase_12
ENSMUSG00000050234	94.64033167	323.1390031	1.6229	1.99E-06	0.00099905	Gja4	chr4	gap_junction_protein_alpha_4
ENSMUSG00000050578	264.4834413	8173.387398	3.4905	2.84E-10	6.51E-07	Mmp13	chr9	matrix_metallopeptidase_13
ENSMUSG00000050808	1652.289958	393.8291786	-1.7781	3.34E-05	0.0094148	Muc15	chr2	mucin_15
ENSMUSG00000052305	380.8432827	41.33505788	-2.5021	8.21E-07	0.00059886	Hbb-bs	chr7	hemoglobin_beta_adult_s_chain

ENSMUSG00000053552	22.97046569	158.9990138	1.9909	0.0003297	0.039595	Ebf4	chr2	early_B_cell_factor_4
ENSMUSG00000056290	16.20111018	80.14673444	1.8239	0.00027237	0.035844	Ms4a4b	chr19	membrane-spanning_4-domains_subfamily_A_member_4B
ENSMUSG00000056328	195.1307524	5.515856127	-2.9008	1.43E-06	0.0008477	Myh1	chr11	myosin_heavy_polypeptide_1_skeletal_muscle_adult
ENSMUSG00000057074	1.897845406	64.30735876	2.7123	7.81E-06	0.0031333	Ces1g	chr8	carboxylesterase_1G
ENSMUSG00000058446	5304.139002	1677.485465	-1.4755	0.00014646	0.023514	Znrf2	chr6	zinc_and_ring_finger_2
ENSMUSG00000059336	3.043856349	33.12808699	2.2439	0.00010718	0.020245	Slc14a1	chr18	solute_carrier_family_14_(urea_transporter)_member_1
ENSMUSG00000060548	423.7727386	102.1408321	-1.7612	4.21E-05	0.011071	Tnfrsf19	chr14	tumor_necrosis_factor_receptor_superfamily_member_19
ENSMUSG00000060639	116.2504129	507.4778327	1.7918	6.81E-05	0.014815	Hist1h4i	chr13	histone_cluster_1_H4i
ENSMUSG00000060981	42.54869512	423.2573868	2.7887	5.82E-10	1.17E-06	Hist1h4h	chr13	histone_cluster_1_H4h
ENSMUSG00000061048	897.1867682	49.32413692	-2.2513	0.0002295	0.031763	Cdh3	chr8	cadherin_3
ENSMUSG00000061353	95.67010567	419.1521158	1.8706	3.70E-06	0.0017466	Cxcl12	chr6	chemokine_(C-X-C_motif)_ligand_12
ENSMUSG00000061462	105.6346548	9.776667158	-2.1757	0.00021906	0.030851	Obscn	chr11	obscurin_cytoskeletal_calmodulin_and_titin-interacting_RhoGEF

ENSMUSG00000061723	729.444135	25.32898253	-3.2152	1.98E-08	2.44E-05	Tnnt3	chr7	troponin_T3_skeletal_fast
ENSMUSG00000061816	310.9525939	2.314961198	-4.225	2.87E-13	1.50E-09	Myl1	chr1	myosin_light_polypeptide_1
ENSMUSG00000064288	22.49879791	102.0335887	1.769	0.00026294	0.035157	Hist1h4k	chr13	histone_cluster_1_H4k
ENSMUSG00000067149	20.11055396	236.6367132	2.257	0.00012556	0.022152	Igj	chr5	immunoglobulin_joining_chain
ENSMUSG00000068245	160.7105701	40.38814347	-1.6874	0.00014822	0.023561	Phf11d	chr14	PHD_finger_protein_11D
ENSMUSG00000068697	58.11149334	1.691325336	-2.9015	1.15E-06	0.00077249	Myoz1	chr14	myozenin_1
ENSMUSG00000069670	25.00802035	0	-2.7474	8.23E-06	0.0032225	Nkain2	chr10	Na+/K+_transporting_ATPase_interacting_2
ENSMUSG00000070469	6.576736783	54.96544739	2.2874	1.83E-05	0.0056107	Adamtsl3	chr7	ADAMTS-like_3
ENSMUSG00000070576	29.50863953	205.7058527	2.3429	2.19E-07	0.00019495	Mn1	chr5	meningioma_1
ENSMUSG00000071723	167.4481304	28.70561891	-1.9766	0.00011194	0.020422	Gspt2	chrX	G1_to_S_phase_transition_2
ENSMUSG00000072941	134.4927454	1194.80721	2.2304	6.35E-05	0.014348	Sod3	chr5	superoxide_dismutase_3_extracellular
ENSMUSG00000073988	144.3099243	25.21490186	-1.9022	0.00031111	0.038422	Ttpa	chr4	tocopherol_(alpha)_transfer_protein
ENSMUSG00000074115	5.764502398	112.5518694	2.3087	0.00015386	0.024219	Saa1	chr7	serum_amyloid_A_1
ENSMUSG00000074665	10.45585219	85.73331316	2.3982	1.62E-06	0.0008957	Bpifb4	chr2	BPI_fold_containing_family_B_member_4

ENSMUSG00000074892	2672.728367	309.5596118	-2.0815	0.00030789	0.038319	B3galt5	chr16	UDP-Gal:betaGlcNAc_beta_13-galactosyltransferase_polypeptide_5
ENSMUSG00000075307	93.94206338	8.810349678	-2.0901	0.00045189	0.049021	Klhl41	chr2	kelch-like_41
ENSMUSG00000076609	45.74178347	851.0081252	2.4902	3.34E-05	0.0094148	Igkc	chr6	immunoglobulin_kappa_constant
ENSMUSG00000076613	3.513636023	82.2127103	2.4639	5.04E-05	0.012271	Ighg2b	chr12	immunoglobulin_heavy_constant_gamma_2B
ENSMUSG00000078495	85.40000803	6.890929591	-2.7078	3.19E-07	0.00026871	Gm13157	chr4	predicted_gene_13157
ENSMUSG00000078503	311.4759695	41.88166013	-2.1854	3.42E-05	0.0094607	Gm13225	chr4	predicted_gene_13225
ENSMUSG00000079243	76.36494829	2.69511394	-2.2145	0.00033048	0.039595	Xirp1	chr9	xin_actin-binding_repeat_containing_1
ENSMUSG00000079259	2.215900988	44.22081097	2.4456	4.82E-05	0.012185	Trim71	chr9	tripartite_motif-containing_71
ENSMUSG00000090066	62.85375996	228.0504422	1.5825	0.0003232	0.039595	1110002E22Rik	chr3	RIKEN_cDNA_1110002E22_gene
ENSMUSG00000092569	30.13941327	138.6994022	1.7968	0.00015996	0.024694	Gm20544	chr18	predicted_gene_20544
ENSMUSG00000093594	39.47787749	4.065840415	-2.1285	0.00025023	0.034337	Gm20707	chr4	predicted_gene_20707
ENSMUSG00000095079	44.42995874	1099.897351	2.645	1.17E-05	0.0040676	Igha	chr12	immunoglobulin_heavy_constant_alpha
